# Multi‐Component Functionalized *Bifidobacterium Longum* Hydrogel for Multi‐Target Integrated Therapy of Colitis‐Associated Anxiety and Depression

**DOI:** 10.1002/advs.76242

**Published:** 2026-06-22

**Authors:** Shuo Zhang, Yujie Zhang, Jiansheng He, Shunlian Li, Qingyan Ma, Qiao Li, Yudan Zhang, Yiyang Wang, Shaobo Ma, Songyan Jin, Chune Li, Xueyong Xie, Hang Zhang, Junze Deng, Xueqin Song, Daocheng Wu, Xiancang Ma, Feng Zhu

**Affiliations:** ^1^ Center for Brain Science & Department of Psychiatry The First Affiliated Hospital of Xi'an Jiaotong University Xi'an China; ^2^ Department of Breast Disease The Affiliated Cancer Hospital of Zhengzhou University & Henan Cancer Hospital Zhengzhou China; ^3^ The Key Laboratory of Biomedical Information Engineering of Ministry of Education School of Life Science and Technology Xi'an Jiaotong University Xi'an China; ^4^ Center for Translational Medicine & Department of Psychiatry The First Affiliated Hospital of Xi'an Jiaotong University Xi'an China; ^5^ Biological Psychiatry International Joint Laboratory of Henan Zhengzhou University Zhengzhou China

**Keywords:** *bifidobacterium longum*, colitis‐associated anxiety and depression, gut‐brain axis, hydrogel, metabolites

## Abstract

Inflammatory bowel diseases (IBDs) are frequently accompanied by anxiety and depression, largely driven by perturbed gut‐brain axis signaling. However, current oral therapies remain constrained by the spatial and functional separation between intestinal inflammation and central nervous system dysfunction. Here, we present a comprehensive gut‐brain dual region integrated therapeutic strategy based on functionalized *Bifidobacterium longum* hydrogel (INPs@BL@Gel), in which baicalin and tyrosine are coordinated with Fe(III) to form infinite coordination polymers (ICPs), coated with inulin, assembled onto *Bifidobacterium longum* (BL), and subsequently encapsulated within a pH‐ and matrix metalloproteinase‐responsive silk fibroin‐gelatin hydrogel. INPs@BL@Gel exhibits high drug‐loading, effective gastric protection, inflammation‐triggered release, and long‐term intestinal colonization. Within the inflamed intestine, BL and components synergistically suppress inflammatory responses, restore gut microbiota homeostasis, and promote intestinal barrier repair through multi‐target integrated therapy. Importantly, BL combined with components markedly enhances the production of beneficial neuroactive metabolites such as homovanillic acid and short‐chain fatty acids, which integratedly regulate neuroinflammation, preserve synaptic function, and facilitate blood‐brain barrier repair via the gut‐brain axis. In vivo studies demonstrate that INPs@BL@Gel not only exerts potent therapeutic efficacy against colitis and effectively alleviates associated depression, but also reshapes the gut microbiota and restores barrier integrity, achieving a remarkable comprehensive therapeutic effect.

## Graphical abstract

A pH/MMP dual‐responsive *Bifidobacterium longum* (BL) hydrogel (INPs@BL@Gel) functionalized with baicalin, tyrosine, and inulin is constructed. It enables gastric protection, inflammation‐targeted release, and prolonged intestinal colonization. By remodeling gut microbiota, elevating beneficial neuroactive metabolites (homovanillic acid, short‐chain fatty acids), repairing the intestinal barrier and blood‐brain barrier, this hydrogel achieves synergistic multi‐target therapy for colitis‐associated anxiety and depression via gut‐brain axis regulation.

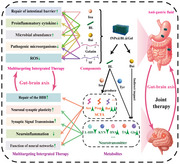

## 1 Introduction

Inflammatory bowel diseases (IBDs) are complex, immune‐mediated disorders characterized by chronic and relapsing inflammation of the gastrointestinal tract, with Crohn's disease (CD) and ulcerative colitis (UC) representing the two major subtypes [1, 2]. Beyond persistent intestinal injury, accumulating epidemiological and clinical evidence indicates that patients with IBDs exhibit a significantly higher prevalence of mental health comorbidities, particularly anxiety and depression, compared with both the general population and individuals with other chronic diseases [3, 4, 5]. These psychological disorders markedly impair quality of life and may further aggravate intestinal inflammation through stress‐related pathways, immune dysregulation, and disturbances in neuroendocrine function [6, 7, 8]. Together, these interactions constitute a bidirectional communication network between the gut and the brain, commonly referred to as the gut‐brain axis, thereby increasing the complexity of long‐term IBD management. Therefore, there is an urgent need to develop therapeutic strategies capable of simultaneously alleviating intestinal inflammation and the anxiety‐ and depression‐like symptoms associated with colitis.

Currently, oral administration remains the primary therapeutic approach for IBDs and their associated anxiety and depression because of its favorable safety profile, high patient compliance, and ease of administration [9]. However, existing oral treatment strategies are limited by the problem of “site separation.” On the one hand, anti‐inflammatory agents and microbial therapies mainly target local intestinal inflammation but exert limited benefits on psychological symptoms [10, 11]. On the other hand, although antidepressants and anxiolytics can regulate central nervous system (CNS) function, they are often inadequate for preventing the onset or recurrence of intestinal inflammation [8, 12]. More importantly, intestinal inflammation and mental disorders are not independent conditions; rather, they constitute a highly interconnected and bidirectional regulatory network mediated through the gut‐brain axis. This complex network involves neural pathways, such as the vagus nerve and enteric nervous system; immune signaling pathways, including the translocation of pro‐inflammatory cytokines across biological barriers; endocrine regulation through the hypothalamic‐pituitary‐adrenal axis; and microbial metabolites, such as short‐chain fatty acids (SCFAs) and neurotransmitter precursors [13, 14, 15]. Chronic intestinal inflammation can initiate or aggravate CNS dysfunction through these mechanisms, whereas psychological disorders may, in turn, amplify inflammatory responses, thereby creating a self‐perpetuating vicious cycle [7, 16]. In addition, intestinal microbiota dysbiosis and impairment of both the intestinal barrier and the blood‐brain barrier (BBB) further compromise the long‐term efficacy of single‐target therapies. Therefore, therapeutic strategies directed toward only a single organ or signaling pathway are unlikely to effectively manage colitis‐associated anxiety and depression.

In recent years, probiotics have attracted considerable attention as oral therapeutic agents for IBDs because of their favorable biosafety profile and their ability to regulate gut microbiota, strengthen the intestinal mucosal barrier, and restore immune homeostasis [17, 18, 19]. To address limitations such as gastrointestinal degradation and poor colonization efficiency, various strategies, including hydrogel encapsulation, genetic engineering, and composite formulations, have been developed. Importantly, the therapeutic effects of probiotics are not confined to the intestinal tract. Probiotic‐derived metabolites can also modulate CNS function through the gut‐brain axis, thereby providing a promising strategy for the integrated treatment of colitis‐associated depression. For example, Wang et al. (2022) developed a light‐responsive *Lactococcus lactis* system capable of interacting with the enteric nervous system (ENS). In this system, glucagon‐like peptide‐1 (GLP‐1) signaling through ENS‐associated GLP‐1 receptors activated neuronal nitric oxide synthase (nNOS), thereby stimulating vagal pathways and modulating CNS activity [20]. More recently, Jia et al. (2024) conducted a large cohort study demonstrating that *Bifidobacterium longum* (BL) can synthesize homovanillic acid (HVA) from tyrosine (Tyr). In a mouse model of depression, HVA selectively enhanced the expression of the presynaptic protein synapsin 1 (SYN1) in the hippocampus, thereby providing experimental evidence for potential mechanisms and therapeutic targets involved in colitis‐associated depression [21]. Collectively, these studies provide preliminary support for the concept of “gut‐targeted, brain‐modulating” therapeutic strategies.

Despite these advances, probiotic‐based systems still exhibit several important limitations that restrict their capacity to achieve coordinated treatment of colitis‐associated depression. (1) Single‐dimensional intervention: Most current studies primarily focus on alleviating intestinal inflammation, while their effects on mental disorders are generally indirect and mediated through microbial metabolites. Multi‐component and multi‐metabolite strategies are rarely explored, and integrated therapeutic designs simultaneously targeting both intestinal and brain functions remain limited. Consequently, these approaches often fail to disrupt the vicious cycle linking intestinal inflammation and psychological disorders [22, 23]. (2) Limited control over metabolite production: Many probiotic systems depend largely on the intrinsic metabolic activity of probiotics, making it difficult to precisely regulate the type and quantity of metabolites produced [24, 25]. Furthermore, most existing studies concentrate on single metabolites, such as HVA or SCFAs, rather than incorporating multiple bioactive metabolites into a unified therapeutic platform [21, 26]. This limitation reduces the consistency, depth, and overall efficacy of interventions for mental disorders. (3) Insufficient engineering integration: Effective management of both IBDs and associated mental disorders typically requires the coordinated action of multiple therapeutic components and biological functions. However, integrating these elements into a single probiotic‐based platform remains highly challenging, as each component must fulfill several functional roles simultaneously. This imposes stringent requirements on probiotic selection, material compatibility, and system assembly. Most currently reported probiotic systems rely on relatively simple formulations and have not achieved a unified multifunctional platform capable of maintaining stability, promoting intestinal colonization, and simultaneously integrating inflammation suppression, barrier repair, microbiota remodeling, and mental health regulation [27, 28, 29]. Collectively, these limitations hinder the ability of existing probiotic‐based strategies to overcome the traditional problem of “site separation” and to provide comprehensive therapeutic intervention for colitis‐associated anxiety and depression.

**SCHEME 1:**
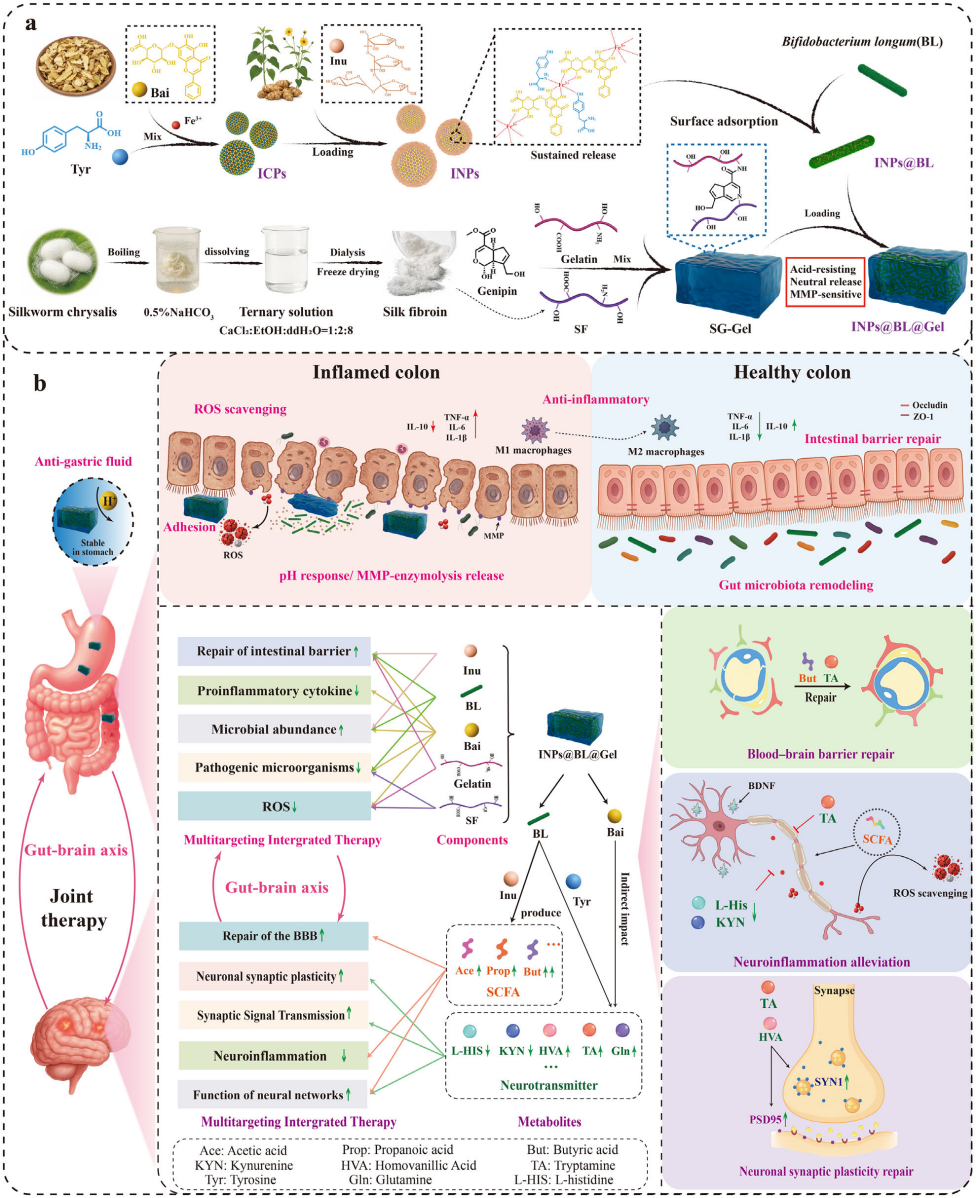
(a) Schematic diagram of the design and preparation of functionalized *Bifidobacterium longum* hydrogel. (b) Exploration of the mechanism of INPs@BL@Gel in treating colitis‐associated anxiety and depression through a dual‐site multi‐target synergistic strategy.

To address these challenges, the present study proposes a comprehensive therapeutic strategy based on the concept of “gut‐brain dual‐region synergistic therapy.” This approach employs a rationally designed probiotic composite system that integrates intestinal barrier repair with gut microbiota remodeling. Through a multi‐component and multi‐target therapeutic framework acting simultaneously on both the gut and brain, the strategy is intended to alleviate intestinal inflammation as well as colitis‐associated anxiety and depression, thereby overcoming the limitations associated with the “site separation” of conventional oral therapies. In addition, the long‐term colonization capacity of probiotics may facilitate sustained remodeling of the gut microbiota and continuous restoration of intestinal barrier integrity [10, 30]. Meanwhile, a variety of key microbial metabolites, including SCFAs and tryptamine‐related molecules, may contribute to the maintenance of BBB function and CNS homeostasis [31, 32]. More importantly, a multifunctional probiotic platform was established in which each component was designed to perform multiple therapeutic roles, thereby enabling the coordinated realization of the aforementioned functions. The overall design, preparation process, and proposed gut–brain dual‐site therapeutic mechanism of INPs@BL@Gel are summarized in Scheme 1a,b.

The strategic design of this study focuses on modulating both the composition and levels of microbial metabolites through controlled interactions between probiotics and functional components. This enables the selective enhancement of beneficial metabolites while suppressing potentially harmful metabolic outputs. BL was selected as the core probiotic strain due to its strong intestinal adaptability and well‐established biosafety profile [21]. Previous studies have demonstrated that BL can restore intestinal barrier integrity by inhibiting pathogen colonization, enhancing epithelial tight junction expression, and regulating immune homeostasis [33, 34]. Moreover, BL can utilize exogenous Tyr to produce neuroactive metabolites, including HVA, and may also influence the activity of other gut microbiota, thereby indirectly promoting the production of butyrate and tryptophan‐related metabolites [21, 33, 35]. Among these metabolites, HVA may modulate CNS function through the gut‐brain axis and contribute to mood regulation by maintaining synaptic homeostasis and regulating neuroautophagy [21]. Tryptamine has also been associated with reduced neuroinflammation and improved synaptic function [36]. In addition, this study incorporates the natural compounds baicalin (Bai) and inulin (Inu) as complementary functional components. Bai exhibits anti‐inflammatory and neuroprotective properties and may contribute to emotional regulation through modulation of monoaminergic neurotransmitter pathways [37]. Inu, a well‐known prebiotic, promotes the selective growth of *Bifidobacterium* and reshapes microbial metabolic networks via cross‐feeding interactions, thereby enhancing short‐chain fatty acid (SCFA) production [38]. Butyrate, a key SCFA, plays an essential role in maintaining both intestinal epithelial integrity and BBB function [39]. Collectively, the combination of BL, Bai, Tyr, and Inu is expected to enhance the production of beneficial neuroactive metabolites and SCFAs while suppressing undesirable metabolic pathways, thereby enabling multi‐target regulation of anxiety‐ and depression‐like symptoms. Meanwhile, BL and the complementary components act synergistically to suppress inflammatory responses, restore gut microbiota homeostasis, and promote intestinal barrier repair through an integrated multi‐target therapeutic strategy.

At the engineering level, a BL‐based composite system (INPs@BL@Gel) was constructed by integrating Bai, Tyr, and Inu with BL through a coordination‐assembly strategy. Bai and Tyr were coordinated with Fe(III) ions to form carrier‐free infinite coordination polymer nanoparticles (ICPs), enabling efficient encapsulation of functional molecules and stimulus‐responsive release under inflammatory conditions. The resulting ICPs were subsequently coated with Inu and assembled onto the surface of BL to obtain the probiotic composite (INPs@BL). To further enhance stability during oral administration, the system was encapsulated within a gelatin‐silk fibroin hydrogel (SG‐Gel), which provides protection against gastric acid and enables responsiveness to pH variations and matrix metalloproteinase (MMP) activity [40]. The resulting INPs@BL@Gel system is designed to achieve effective protection and targeted release at inflammation‐associated sites within the gastrointestinal tract. This engineering strategy not only implements the proposed therapeutic concept but also fully leverages the multifunctional properties of each component, ensuring that no single constituent is restricted to a single role. As a result, a structurally streamlined yet functionally highly integrated INPs@BL@Gel platform is obtained.

In vivo and in vitro experiments confirmed the exceptional biological activity and therapeutic efficacy of INPs@BL@Gel. Compared with the control group, its survival rate in gastric acid environments increased by approximately 56.87‐fold, while its colonization in the inflamed intestine increased by about 10.58‐fold, thereby overcoming common challenges in oral probiotic delivery. INPs@BL@Gel significantly promoted intestinal inflammation resolution, as evidenced by increased colonic goblet cell abundance and restoration of colon length to near‐normal levels. It also modulated the expression of pro‐inflammatory cytokines, including TNF‐α, IL‐1β, and IL‐6, while upregulating the anti‐inflammatory cytokine IL‐10. Behavioral assessments revealed significant improvements in spontaneous activity, anxiety‐like behavior, and reward responsiveness in treated mice, accompanied by reduced immobility time in both the tail suspension test (TST) and forced swimming test. Mechanistic investigations indicated that the system reversed gut dysbiosis, restored SCFA‐producing bacteria, and corrected key metabolite imbalances, thereby contributing to BBB repair and the regulation of synaptic plasticity. Overall, this study provides a new framework for oral, integrated intervention in colitis‐associated depression and offers broader insights into probiotic engineering for gut‐brain axis‐related diseases.

## 2 Results and Discussion

### 2.1 Preparation and Characterization of INPs and SG‐Gel

To increase the drug loading capacity of the therapeutic agent and ensure its effective release at the site of enteritis, we constructed ICPs by coordinating Bai, Tyr, and Fe^3+^ ions. This infinite coordination polymer exhibits a drug loading capacity approaching 100% and enables drug release in the inflammatory microenvironment. Subsequently, Inu, a polysaccharide, was coated onto the surface. Given the structural similarity between Inu and Bai, intermolecular hydrogen bonding and hydrophobic interactions were leveraged to facilitate the deposition of Inu onto the surface of the ICP nanoparticles via π‐π stacking interactions. Both the infinite coordination assembly and π‐π stacking represent carrier‐free, self‐assembled drug delivery systems characterized by high drug loading efficiency and stimuli‐responsive release at inflammatory sites. Finally, INPs@BL@Gel was obtained by coating the ICP nanoparticles onto the bacterial system and further encapsulating them within a hydrogel composed of SG‐Gel. The SG‐Gel hydrogel exhibits excellent acid resistance and inflammation‐responsive release behavior, thereby meeting the requirements for multitargeted, integrated therapy of intestinal inflammation.

**FIGURE 1:**
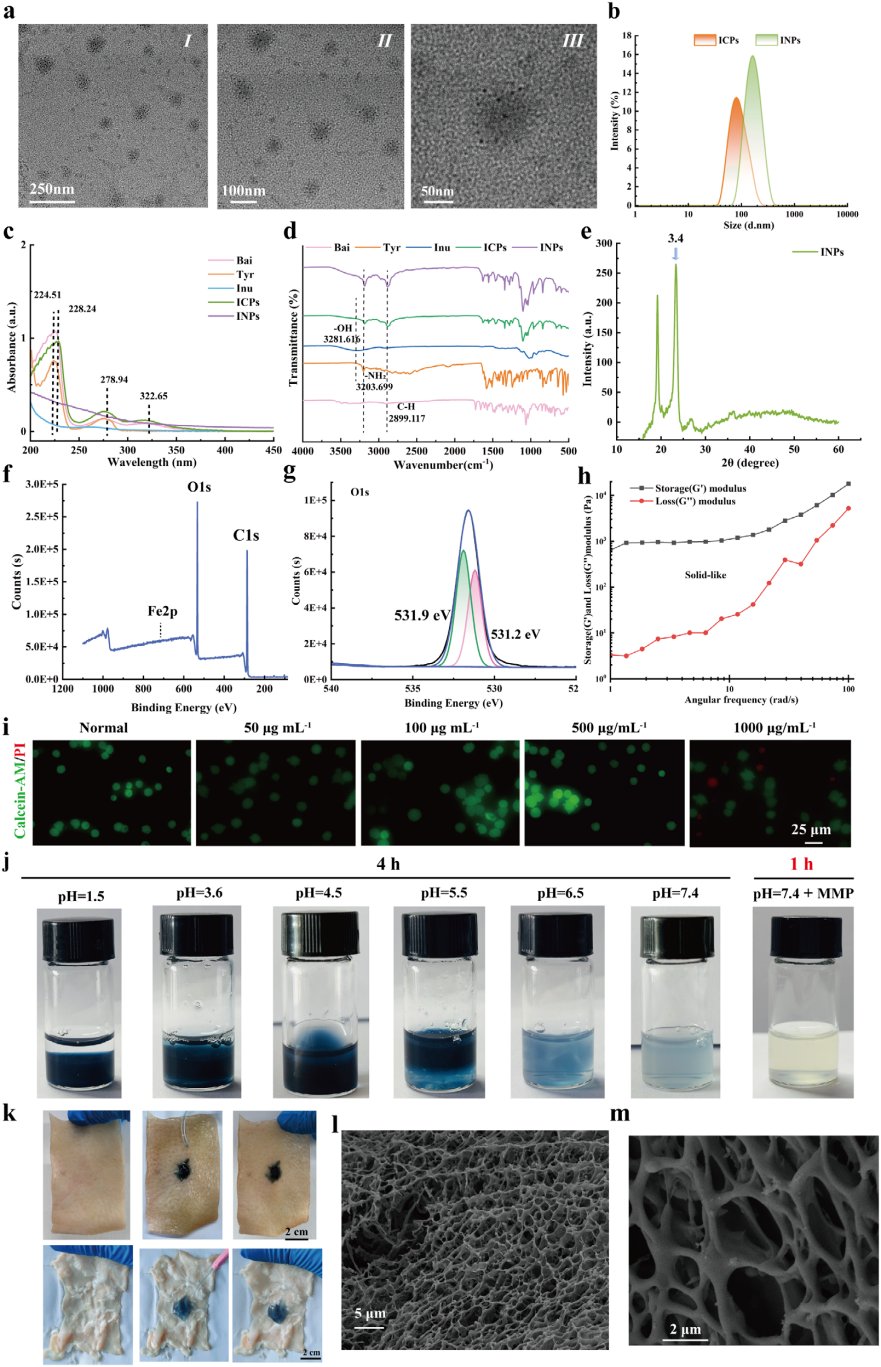
Characterization of INPs and SG‐Gel. (a) Representative TEM images of INPs (with different magnifications). (b) Size distribution of ICPs and INPs. (c, d) UV–vis absorption spectra and FTIR spectra of various compounds in INPs. (e) XRD pattern of INPs. (f, g) The full‐spectrum graph of XPS in INPs and the peak‐spectrum graph of the O 1s element. (h) Rheological properties of SG‐Gel. (i) The cytotoxicity of INPs. (j) pH‐ and MMP‐responsive degradation behavior of SG‐Gel. (k) The porcine skin adhesion of SG‐Gel (Top); the intestinal mucus adhesion property of SG‐Gel (bottom). (l, m) Representative SEM images of SG‐Gel (with different magnifications).

**FIGURE 2:**
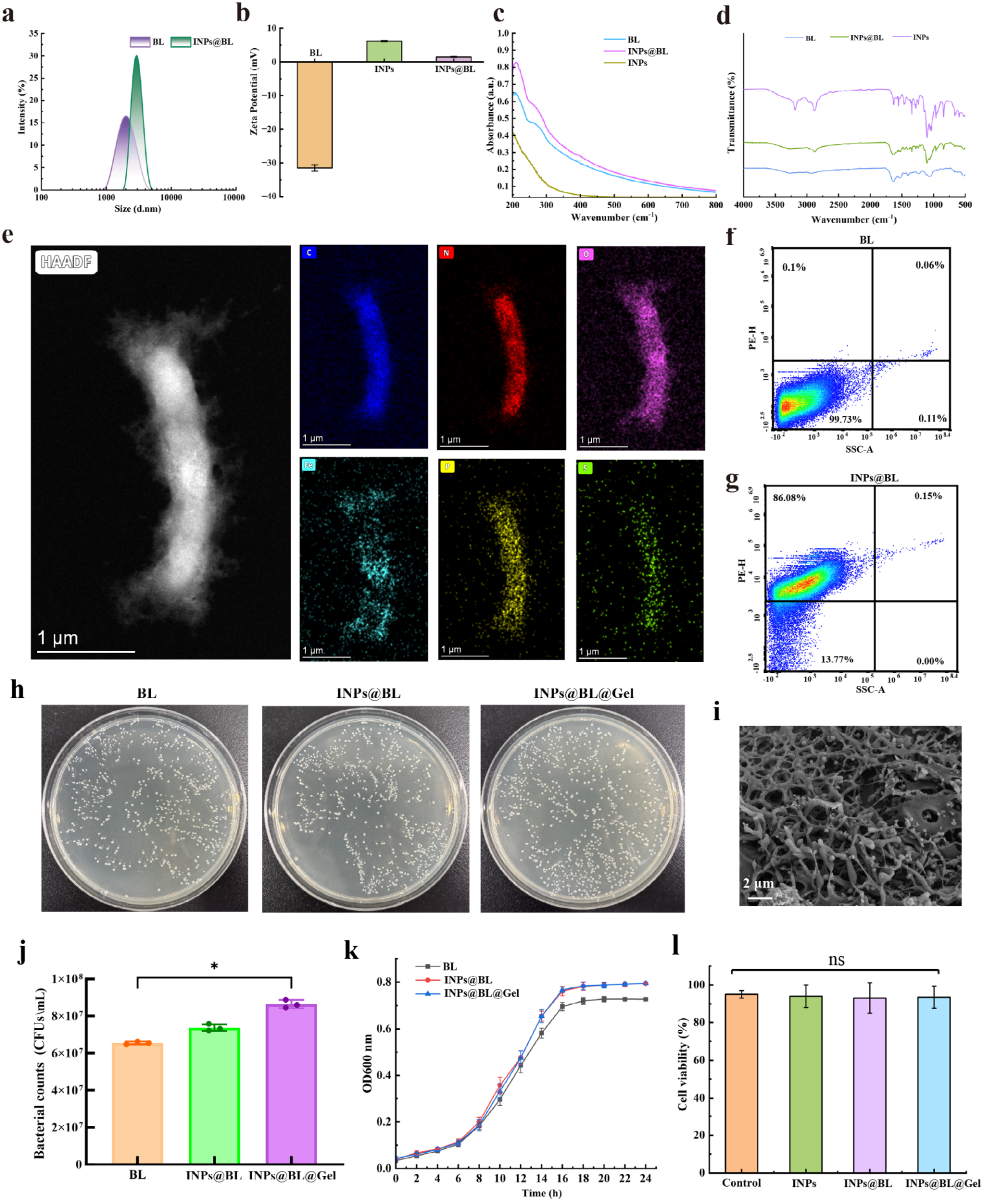
Characterization of INPs@BL@Gel. (a) The particle sizes of BL and INPs@BL. (b‐d) The zeta potential, ultraviolet spectra, and infrared spectra of BL, INPs, and INPs@BL. (e) HAADF‐STEM images and EDS spectra of INPs@BL. (f, g) Flow cytometry analysis of the coating efficiency of INPs on BL. (h, j) Photographs and corresponding counts of bacterial colonies formed on TPY agar plates. (i) Representative SEM image of INPs@BL@Gel. (k) Growth curves of BL, INPs@BL, and INPs@BL@Gel in TPY medium were evaluated by OD600, n = 3. (l) Cell viability of NCM 460 cells treated with different drug groups (BL, INPs@BL, and INPs@BL@Gel) for 2 h. Data are expressed as Mean ± standard deviation (Mean ± SD), n = 3. Comparisons among multiple groups were performed using one‐way analysis of variance (ANOVA), followed by Tukey's post hoc test for pairwise comparisons (^*^ *p* < 0.05, ^**^ *p* < 0.01, ^***^ *p* < 0.001, and ^****^ *p* < 0.0001).

As shown in Figure 1a, transmission electron microscopy (TEM) revealed that INPs exhibit a spherical morphology with an average particle size of 85 ± 11.5 nm. The TEM images clearly demonstrate a core‐shell structure, in which the dark core corresponds to the ICP framework and the lighter shell represents the Inu layer (Figure S1). In addition, the formation of these INP nanoparticles via fructan‐coated ICPs was confirmed using fluorescence labeling (Figure S3). Dynamic light scattering (DLS) measurements further verified the formation of INPs, showing a hydrodynamic diameter of 113 ± 13.2 nm, which is larger than that of the ICPs alone (Figure 1b). The INPs also exhibited a positive surface charge (Figure 2b). To evaluate nanoparticle stability, INPs were stored in phosphate‐buffered saline (PBS) for 60 days. During this period, their hydrodynamic diameter remained stable at approximately 118 nm (Figure S2), with no observable aggregation or degradation, indicating excellent structural stability under physiological conditions. X‐ray diffraction (XRD) analysis revealed a prominent diffraction peak at d = 3.4 Å, corresponding to the characteristic interplanar spacing of aromatic stacking interactions, indicating that INP self‐assembly is primarily driven by π‐π interactions (Figure 1e). UV–vis absorption spectra of Bai, Tyr, and their Fe^3+^ complexes further confirmed the successful formation of ion‐coordinated ICPs. Upon coordination, the absorption band of Bai shifted from 224.51 to 228.24 nm, while the characteristic peaks of Bai at 278.94 nm and Tyr at 322.65 nm were retained in the ICPs (Figure 1c). Fourier‐transform infrared (FTIR) spectroscopy provided additional evidence of coordination interactions. The C‐H stretching vibration peak at 2899.12 cm^−1^ on the benzene ring exhibited a slight shift and intensity change after ICP formation, suggesting that aromatic hydrogen environments were affected by coordination. In the case of Tyr, the amino group (‐NH_2_) band at 3203.70 cm^−1^ showed a partial red shift following INP formation, further supporting coordination bond formation (Figure 1d). X‐ray photoelectron spectroscopy (XPS) analysis was performed to further validate these findings. As shown in Figure 1f and Table S2, the nanoparticles contain C, O, and Fe elements. The Fe 2p binding energies of the nanoparticles (710.80 and 725.16 eV), compared with those of FeCl_3_, were deconvoluted into four peaks at 709.10, 711.58, 721.90, and 724.20 eV. The observed shifts to lower binding energies relative to FeCl_3_ indicate that Fe(III) accepts lone‐pair electrons from coordinating atoms, confirming successful coordination. In addition, the O 1s binding energies of the nanoparticles (531.9 and 531.2 eV) were higher than those of Bai alone (531.2 and 529.3 eV) and Tyr alone (531.5 and 529.6 eV), further supporting ICP formation (Figure 1g). The cytotoxicity of the samples was evaluated in intestinal epithelial cells. Cell viability remained largely unchanged across a broad concentration range of INPs, with only a minor degree of cell death observed at concentrations exceeding 1 mg mL^−1^. These results indicate that INPs exhibit low cytotoxicity toward intestinal epithelial cells, with appreciable effects only at high doses (Figure 1i). Quantitative analysis further demonstrated that even at 1000 µg mL^−1^, cell viability remained above 80% (Figure S4). Overall, these physicochemical and biological characterization results demonstrate that INPs possess favorable stability and biocompatibility for subsequent in vivo applications.

For oral and intragastric administration, a hydrogel with appropriate flowability and gelation properties is essential for effective protection and delivery. To this end, a dark‐blue SG‐Gel was prepared by crosslinking silk fibroin (SF) extracted from silkworm cocoons with gelatin in the presence of genipin. Rheological analysis of SG‐Gel showed that the storage modulus (G′), representing elastic behavior, remained consistently higher than the loss modulus (G″), representing viscous behavior, across the entire range of angular frequencies (Figure 1h). As angular frequency increased, G′ remained relatively stable with a slight upward trend, whereas G″ gradually increased, indicating that the hydrogel exhibits predominantly elastic behavior at low frequencies and transitions toward more viscous behavior at higher frequencies. The stability of SG‐Gel under different pH conditions was further evaluated by incubating it in buffer solutions of varying pH values. As shown in Figure 1j and Figure S5a, under strongly acidic conditions (pH 1.5), SG‐Gel remained largely intact after 4 h, with a residual gel mass of approximately 2.205 ± 0.05 g. As the pH increased, the gel gradually degraded, with mass reductions of 15.65%, 25.12%, 35.83%, and 78.59% at pH 3.6, 4.5, 5.5, and 6.5, respectively. At pH 7.4, only trace gel residues were observed, with the mass decreasing to 0.024 ± 0.04 g, indicating that SG‐Gel is stable under strongly acidic conditions but progressively degrades as the environment becomes less acidic. Notably, in the presence of MMP, SG‐Gel underwent complete degradation within 1 h at pH 7.4, accompanied by a color change from deep blue to transparent (Figure 1j and Figure S5b). BL@Gel and INPs@BL@Gel exhibited similar degradation behavior, although INPs@BL@Gel appeared slightly more turbid (Figure S6a,b). These results indicate that the system is stable under acidic conditions but degrades more rapidly in inflammatory microenvironments rich in MMPs. Subsequently, the degradation behavior of SG‐Gel was assessed in simulated gastric fluid (SGF) and simulated intestinal fluid (SIF). As shown in Figure S7, SG‐Gel remained largely intact in SGF over 4 h, with minimal degradation. In contrast, it gradually disintegrated in SIF, and its degradation rate in MMP‐containing SIF was approximately four times higher than that in SIF alone, demonstrating dual responsiveness to neutral pH and MMPs. This property enables SG‐Gel to degrade selectively in the intestinal environment and release its payload at the target site. The mucoadhesive properties of SG‐Gel were further evaluated using porcine skin and porcine colonic tissue. As shown in Figure 1k, SG‐Gel adhered firmly to porcine skin tissue without slipping, even when the tissue was held vertically for extended periods. Similarly, strong adhesion was observed on porcine colonic mucosa, indicating excellent mucoadhesive performance. These findings suggest that SG‐Gel can achieve prolonged intestinal retention following intragastric administration. Finally, scanning electron microscopy (SEM) revealed that SG‐Gel possesses a three‐dimensional porous network structure, which is favorable for loading and encapsulating therapeutic agents (Figure 1l,m).

### 2.2 Characterization of INPs@BL@Gel

BL is a widely utilized probiotic known for its excellent biocompatibility and reported anti‐inflammatory and gut microbiota‐modulating effects, making it a promising candidate for the treatment of gastrointestinal disorders and associated neurobehavioral symptoms. Previous studies have shown that BL can help restore the intestinal mucosal barrier and regulate immune responses. In addition, its metabolites have been linked to neuroprotective effects and improved synaptic plasticity, which may contribute to alleviating colitis‐associated depressive‐like behaviors. Based on these findings, *BL* ATCC BAA‐999 was selected as the therapeutic strain, and INPs were engineered onto its surface to enhance its therapeutic potential. INPs are rich in hydroxyl groups and amino acid‐derived structural motifs, which underpin their strong adhesive properties. These features enable INPs to adhere effectively to biological interfaces such as cells, tissues, and mucosal membranes, thereby facilitating stable attachment to the bacterial surface. By coating BL with INPs to form a multifunctional surface layer, the therapeutic performance of the bacteria was significantly enhanced, leading to the successful construction of the INPs@BL composite system.

As shown in Figure 2a, the hydrodynamic diameter of BL increased slightly after loading with INPs, indicating successful surface modification of the bacteria. SEM further revealed that the bacterial surface became noticeably rough following INP modification, in clear contrast to the smooth surface of native BL (Figure S11a,b). Zeta potential measurements confirmed that BL and INPs carry opposite surface charges, highlighting the role of electrostatic interactions in their assembly process (Figure 2b). UV–vis spectroscopy provided additional evidence for the formation of INPs@BL. The spectrum of INPs@BL retained the characteristic absorption peak of BL at 275 nm and exhibited an additional band at 205 nm, which can be attributed to the combined contributions of INPs and BL (Figure 2c). FTIR analysis further corroborated the successful assembly of INPs@BL. Specifically, the band at 3263.51 cm^−1^ in INPs@BL overlapped with that of BL, while the peak at 2899.12 cm^−1^ corresponded to that of INPs, indicating the coexistence of spectral features from both components in the hybrid system (Figure 2d). For comparison, both SF and gelatin, as protein‐based materials, exhibit characteristic amide I (≈1650 cm^−1^), amide II (≈1540 cm^−1^), and amide III (≈1240 cm^−1^) absorption bands. In SG‐Gel (silk fibroin‐gelatin hydrogel), the positions and intensities of these bands differ from those of SF and gelatin alone, suggesting structural interactions between the two protein components (Figure S8). In addition, broad peaks around ≈3300 cm^−1^ correspond to O‐H and N‐H stretching vibrations arising from proteins and water molecules, with the broadened signal in SG‐Gel reflecting overlapping contributions from both polymers. High‐angle annular dark‐field scanning transmission electron microscopy (HAADF‐STEM) elemental mapping demonstrated a uniform distribution of Fe on the bacterial surface, confirming successful coating of the coordination‐polymer nanomedicine layer onto BL (Figure 2e). To further verify the presence of INPs on the bacterial surface, INPs were labeled with phycoerythrin (PE), and fluorescence intensity was quantified by flow cytometry. A distinct PE signal was detected in INPs@BL, confirming successful coating, with an estimated surface coverage of approximately 85% (Figure 2f,g). To more directly visualize the coating of INPs on BL, BL was labeled with DAPI, and INPs were labeled with rhodamine B. As shown in Figure S9a–c, extensive coverage of INPs on the bacterial surface was observed. Subsequently, INPs@BL were encapsulated within the hydrogel to obtain INPs@BL@Gel. SEM cross‐sectional imaging confirmed that the hydrogel matrix effectively embedded INPs@BL (Figure 2i). To assess bacterial viability after formulation, INPs@BL and INPs@BL@Gel were plated on agar following exposure to neutral pH and MMP‐triggered gel disintegration. After 48 h, colony counts were maintained or slightly increased, indicating that neither INP coating nor hydrogel encapsulation adversely affected bacterial viability (Figure 2h). In addition, in the absence of hydrogel protection, INPs@BL remained relatively stable in bile salt and mucin solutions for up to 4 h (Figure S10).

We next assessed whether INPs and the hydrogel affected BL proliferation. As shown in Figure 2k, the growth curves of INPs@BL and INPs@BL@Gel closely followed that of unmodified BL over the same incubation period, indicating that neither surface coating nor encapsulation adversely affected bacterial proliferation. Quantitative colony counting further supported this observation. INPs@BL@Gel reached 8.60 × 10^7^ CFU mL^−1^, INPs@BL reached 7.35 × 10^7^ CFU mL^−1^, and BL reached 6.55 × 10^7^ CFU mL^−1^, suggesting that surface modification and hydrogel encapsulation did not inhibit bacterial growth and may have modestly enhanced it (Figure 2j). Overall, bacterial counts in the INPs@BL and INPs@BL@Gel groups were 1.12‐fold and 1.31‐fold higher than in the BL‐only group, respectively. This slight enhancement in proliferation may be attributed to BL's potential utilization of Inu, as well as gelatin and SF components of the hydrogel, as fermentable substrates that could support bacterial growth.

In SGF, the BL group decreased to approximately 4.22 × 10^5^ CFU mL^−1^ after 4 h (Figure S12a,b). In contrast, bacterial viability in SGF was increased by 7.76‐fold for INPs@BL and by 56.87‐fold for INPs@BL@Gel compared with BL. In SIF, INPs@BL and INPs@BL@Gel exhibited 1.59‐fold and 10.58‐fold higher viability than BL after 4 h, respectively (Figure S12c,d). These results demonstrate that SG‐Gel significantly enhances bacterial survival under both gastric and intestinal conditions, thereby facilitating the accumulation of INPs@BL at sites of intestinal injury. Finally, cytotoxicity assays indicated that INPs, INPs@BL, and INPs@BL@Gel all exhibit good biocompatibility (Figure 2l).

### 2.3 Intracellular and Extracellular Antioxidant Activities of INPs@BL@Gel

Inflammation in the colon and brain is often associated with elevated levels of intra‐ and extracellular oxidative stress. This, in turn, exacerbates disease progression by disrupting the intestinal barrier and the BBB, promoting inflammatory responses, and inducing cellular apoptosis. Therefore, mitigating oxidative stress is essential for the treatment of colitis‐associated depression.

**FIGURE 3:**
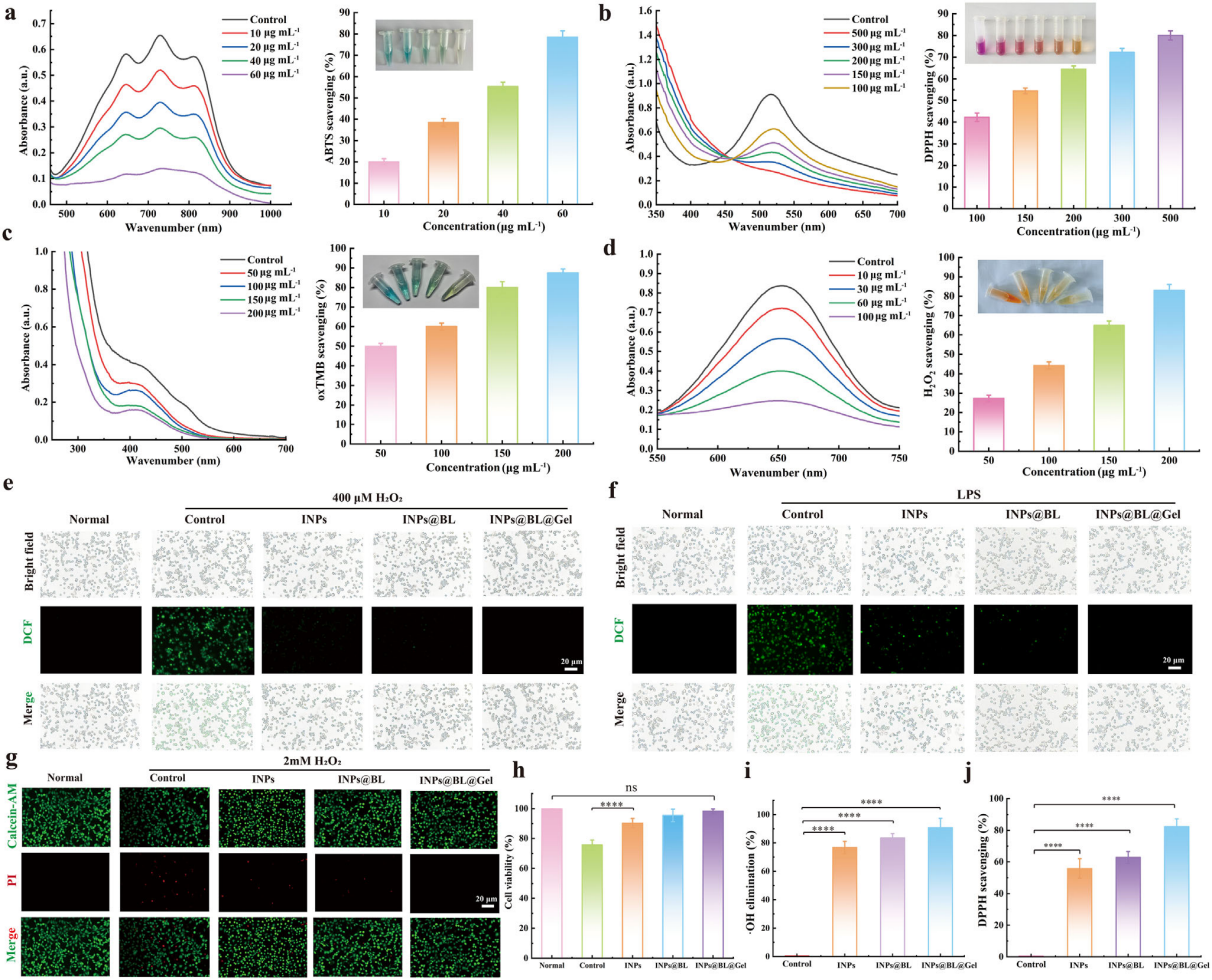
Determination of intracellular and extracellular antioxidant activity of INPs@BL@Gel. (a) The scavenging ability of ABTS free radicals at different INPs concentrations and the corresponding color changes. (b) The free radical scavenging ability of DPPH and the corresponding color changes at different INPs concentrations. (c) TMB was used to detect the scavenging ability of different INPs concentrations for •OH. (d) H_2_O_2_ was used to detect the scavenging ability of different INPs concentrations for O_2_•. (e) The intracellular ROS level in NCM 460 cells was detected after exogenous H_2_O_2_ treatment under a fluorescence microscope. (f) The intracellular ROS level in NCM 460 cells was detected after endogenous LPS induction under a fluorescence microscope. (g) Calcein‐AM/PI fluorescence images of NCM 460 cells treated with 2 mM H_2_O_2_ and various materials for 4 h. Red fluorescence represents dead cells and green fluorescence represents live cells, and activity statistics (h). (i, j) In vitro clearance ability of DPPH and •OH by different material treatment groups. Data are expressed as Mean ± SD, n = 3. Comparisons among multiple groups were performed using one‐way ANOVA, followed by Tukey's post hoc test for pairwise comparisons (^*^ *p* < 0.05, ^**^ *p* < 0.01, ^***^ *p* < 0.001, and ^****^ *p* < 0.0001).

To evaluate the antioxidant capacity of INPs, UV–vis spectrophotometry was used to assess their ability to scavenge various free radicals. As shown in Figure 3a,b, INPs effectively scavenged ABTS (2,2′‐azino‐bis (3‐ethylbenzothiazoline‐6‐sulfonic acid)) and DPPH (1,1‐diphenyl‐2‐picrylhydrazyl) radicals in a concentration‐dependent manner. As the concentration of INPs increased, the levels of both radicals gradually decreased. Consistent with these results, the blue‐green ABTS solution faded to colorless upon incubation with INPs, with absorbance measured at 734 nm. Similarly, the purple DPPH solution gradually turned pale yellow, with absorbance measured at 519 nm.

Furthermore, the overall antioxidant activity of INPs under oxidative stress conditions was evaluated using TMB (3,3′,5,5′‐tetramethylbenzidine) oxidation and H_2_O_2_ scavenging assays. As shown in Figure 3c,d, the removal efficiency of both TMB and H_2_O_2_ increased in a concentration‐dependent manner as the concentration of INPs increased. Correspondingly, the TMB reaction system changed from blue to light yellow, while the H_2_O_2_ assay system shifted from orange‐red to pale yellow. These results demonstrate that INPs possess broad‐spectrum antioxidant activity and can effectively scavenge reactive oxygen species (ROS), thereby alleviating oxidative stress‐induced cellular damage. Based on these findings, a concentration of 150 µg mL^−1^ was selected for subsequent experiments, and INPs@BL and INPs@BL@Gel were prepared using an equivalent INPs dosage.

Additionally, 2′,7′‐dichlorofluorescein diacetate (DCFH‐DA) was employed as a fluorescent probe to evaluate the intracellular ROS‐scavenging activity of INPs, INPs@BL, and INPs@BL@Gel. As shown in Figure 3e,f, INPs significantly reduced intracellular ROS levels in NCM460 cells under both endogenous oxidative stress (induced by LPS) and exogenous oxidative stress (induced by H_2_O_2_). These results indicate that INPs effectively attenuate intracellular oxidative burden and protect cells from oxidative damage. Notably, INPs@BL and INPs@BL@Gel exhibited stronger ROS‐scavenging effects than INPs alone. This enhanced activity may be attributed to Bai, which can inhibit the formation of reactive oxygen and nitrogen species and neutralize free radicals via electron or hydrogen atom donation. By interrupting radical‐mediated chain reactions, Bai suppresses the amplification of oxidative stress. In addition, Bai can directly donate hydrogen atoms to reactive radicals, further contributing to cellular protection against oxidative injury.

In vitro antioxidant assays consistently demonstrated that INPs, INPs@BL, and INPs@BL@Gel (at an equivalent drug dose) exhibit strong free radical scavenging activity against DPPH and hydroxyl radicals (•OH) (Figure 3i,j). Among these formulations, INPs@BL@Gel showed the highest scavenging efficiency, with removal rates of 82.3% for DPPH and 91.0% for •OH. Live/dead staining further confirmed the protective effects of these formulations against oxidative injury. As shown in Figure 3g, treatment with INPs, INPs@BL, and INPs@BL@Gel effectively alleviated H_2_O_2_‐induced damage in NCM460 cells. Quantitative analysis revealed that exposure to 2 mM H_2_O_2_ reduced cell viability to 74.5%, whereas all treatment groups maintained viability above 90.0% (Figure 3h). Taken together, these findings demonstrate that INPs possess pronounced free radical scavenging and antioxidant activity. The synergistic effects of BL and the hydrogel further enhance these properties, improving resistance to oxidative stress, reducing intracellular ROS accumulation, and effectively protecting intestinal epithelial cells from oxidative damage.

### 2.4 Mucoadhesive Properties of INPs@BL@Gel

Stable and long‐term intestinal colonization of probiotics is crucial for achieving sustained therapeutic benefits. SG‐Gel is designed to form a viscous network within the intestine and adhere to the mucus layer, thereby strengthening contact with the intestinal wall and prolonging the residence time of the therapeutic payload at the target site. To evaluate the mucosal adhesion of INPs@BL@Gel in inflamed colonic tissue, fluorescently labeled INPs were co‐incubated with freshly excised intestinal segments from mice with colitis.

**FIGURE 4:**
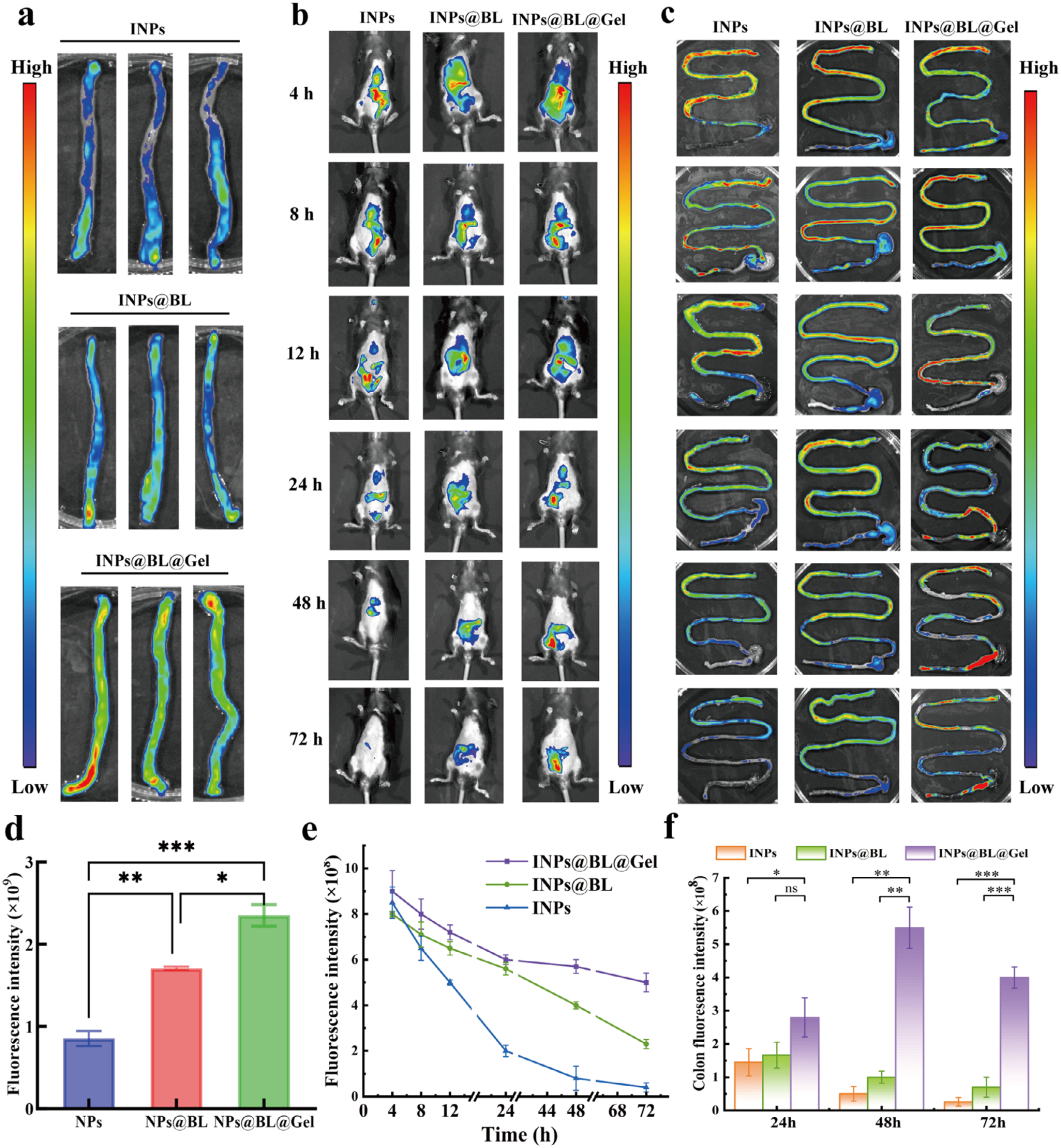
Mucosal adhesion ability of INPs@BL@Gel. (a) In vitro colonic fluorescence images after incubation with different drugs (INPs, INPs@BL, and INPs@BL@Gel) marked with IR780. (b, c) Fluorescence images of live mice and intestinal tracts in vivo treated with different drug groups at different time points. (d) Fluorescence intensity statistics of the colon in vitro. (e) Fluorescence intensity attenuation curve in mice. (f) Fluorescence intensity of the colon of mice at different time points. The High/Low labels indicate the relative fluorescence intensity on the pseudocolor scale, where red/yellow represents higher fluorescence intensity and blue/purple represents lower fluorescence intensity. Data are expressed as Mean ± SD, with n = 3. Comparisons among multiple groups were performed using one‐way ANOVA, followed by Tukey's post hoc test for pairwise comparisons (^*^ *p* < 0.05, ^**^ *p* < 0.01, ^***^ *p* < 0.001, and ^****^ *p* < 0.0001).

As shown in Figure 4a, the fluorescence intensity in the INPs@BL group was higher than that in the INPs group, indicating that probiotics promoted particle retention in the intestinal tissue. Notably, the INPs@BL@Gel group exhibited significantly stronger fluorescence than both the INPs and INPs@BL groups, demonstrating that the hydrogel further enhanced the adhesion of particles and BL to the intestinal mucosa. Fluorescence quantification supported these observations, confirming that SG‐Gel significantly improved particle accumulation within intestinal tissue (Figure 4d).

To more precisely assess material distribution, mice were euthanized at predetermined time points, and their intestines were harvested for ex vivo fluorescence imaging. As shown in Figure 4c,f, at 48 h, the fluorescence intensity in the INPs@BL@Gel group was 11.02‐fold and 5.53‐fold higher than that in the INPs and INPs@BL groups, respectively. By 72 h, fluorescence intensity in the INPs@BL@Gel group remained markedly elevated, being 15.38‐fold and 5.71‐fold higher than that in the INPs and INPs@BL groups, respectively. These results collectively demonstrate that incorporation of BL and SG‐Gel significantly prolongs gastrointestinal retention and enhances sustained localization of the therapeutic system.

Further fluorescence imaging was performed to assess material distribution in major visceral organs. As shown in Figure S13, no detectable fluorescence signals were observed in the heart, liver, spleen, lungs, or kidneys, suggesting that the materials are primarily retained and metabolized within the gastrointestinal tract after oral administration, with minimal systemic distribution to peripheral organs. Notably, the fluorescence intensity in the INPs@BL and INPs@BL@Gel groups increased to 2.13‐fold and 2.65‐fold, respectively, compared with the INPs group (Figure 4d). In vivo fluorescence imaging further showed that fluorescence intensity gradually decreased over time in all groups (Figure 4b,e). However, the INPs@BL@Gel group consistently exhibited significantly higher fluorescence intensity at each time point than the INPs and INPs@BL groups, and displayed a markedly slower decline, maintaining strong fluorescence signals even at 72 h. Quantitative fluorescence decay analysis confirmed that the INPs group exhibited a rapid decrease in fluorescence within 24 h, whereas the INPs@BL and INPs@BL@Gel groups showed substantially slower decay rates. Among them, the INPs@BL@Gel group demonstrated the highest fluorescence intensity at all time points and the slowest clearance rate. These findings suggest that BL and SG‐Gel effectively prolong intestinal retention of the nanoparticles. This effect is likely attributable to the strong mucosal adhesion and colonization capacity of BL, as well as the inflammation‐responsive retention and release behavior of the hydrogel, which together enhance localized therapeutic accumulation in the intestine.

### 2.5 Therapeutic Efficacy of INPs@BL@Gel Against DSS‐Induced Colitis

**FIGURE 5:**
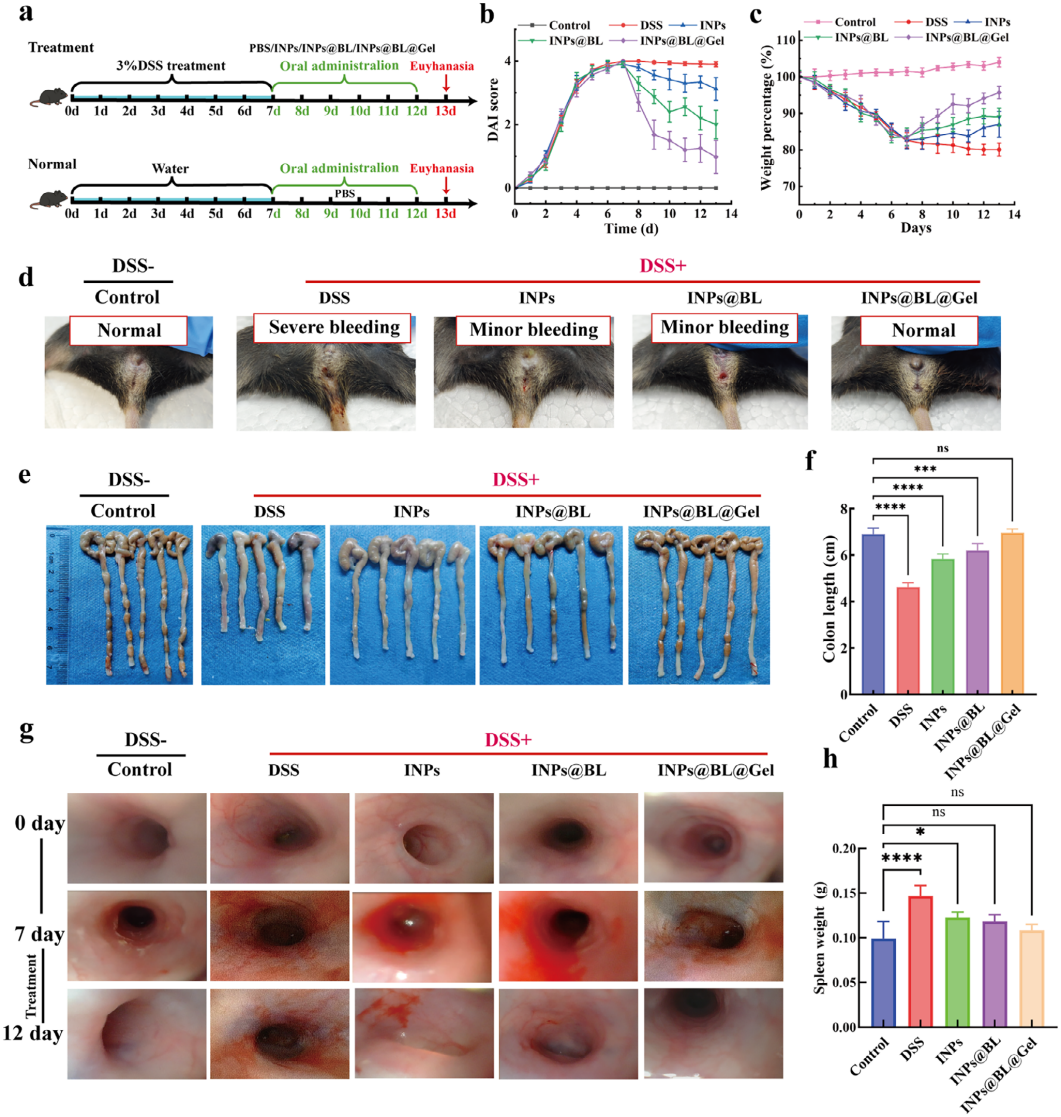
The treatment effect of INPs@BL@Gel on colitis. (a) Schematic diagram of modeling and treatment process. (b) Changes in the DAI of mice treated with different drug groups. (c) Changes in the body weight percentage of mice during the experiment. (d) Representative photos of the anus of each group of mice. (e) Photographs of the colon and (f) the statistics of colon lengths for each group of mice. (g) Endoscopic images of the colon in each group of mice. (h) Quantitative analysis of spleen weight in different groups of mice. Data are expressed as Mean ± SD, with n = 5. Comparisons among multiple groups were performed using one‐way ANOVA, followed by Tukey's post hoc test for pairwise comparisons (^*^ *p* < 0.05, ^**^ *p* < 0.01, ^***^ *p* < 0.001, and ^****^ *p* < 0.0001).

To evaluate the in vivo therapeutic potential of INPs@BL@Gel, a comprehensive assessment was conducted in a mouse model of colitis induced by 3% dextran sulfate sodium (DSS). Mice were randomly assigned to five groups and, beginning on day 7 after DSS administration, received daily treatments with PBS, INPs, INPs@BL, or INPs@BL@Gel for five consecutive days (Figure 5a). A healthy control group was included and administered PBS via gavage over the same period. Following the treatment phase, mice were euthanized, and tissues were collected for subsequent analyses.

Throughout the study, body weight, colonic condition, and disease activity index (DAI), including weight loss, stool consistency, and rectal bleeding, were carefully monitored. As shown in Figure 5b,c, and Table S3, DSS‐treated mice exhibited significant body weight loss and a marked increase in DAI, indicating severe colitis. In contrast, all treatment groups showed varying degrees of disease alleviation. Notably, mice treated with INPs@BL@Gel displayed a clear rebound in body weight during the later treatment stage, accompanied by a significant reduction in DAI, approaching levels observed in healthy controls. Additionally, DSS exposure induced prominent clinical symptoms, including rectal bleeding and diarrhea, whereas INPs@BL@Gel treatment most effectively alleviated these manifestations (Figure 5d). On day 13, mice were euthanized, and colonic tissues were collected for further analyses. Colon shortening is a well‐established indicator of colonic inflammation (Figure 5e). Quantitative measurements showed that, compared with the control group (6.90 cm), colon length was reduced by 33.04%, 15.36%, and 10.14% in the DSS (4.62 cm), INPs (5.84 cm), and INPs@BL groups (6.20 cm), respectively. In contrast, the colon length in the INPs@BL@Gel group (6.96 cm) was comparable to that of healthy controls, with no significant difference (Figure 5f). Spleen weight near‐complete recovery analysis revealed marked splenomegaly in DSS‐induced mice, with spleen weight approximately 1.50‐fold higher than that of healthy mice. By contrast, no significant splenic enlargement was observed in the INPs@BL@Gel group, which was comparable to the control group (Figure 5h and Figure S14). In addition, a colonoscopy was performed to monitor mucosal changes throughout the treatment period. As shown in Figure 5g, DSS‐treated mice exhibited severe inflammatory features, including mucosal bleeding, disrupted vascular patterns, mucosal opacity, and adhesions. Notably, INPs@BL@Gel treatment markedly restored mucosal architecture, with colonoscopic findings closely resembling those of healthy controls. Compared with a previous study by the Sung‐Kwon Moon group reporting only 19.30% recovery in colon length (approximately 4.6 cm after treatment), our INPs@BL@Gel group achieved near‐complete recovery (∼6.9 cm), which was not significantly different from the normal group [41]. This outcome highlights the strong therapeutic efficacy of INPs@BL@Gel.

Collectively, in the DSS‐induced colitis model, INPs, INPs@BL, and INPs@BL@Gel all exhibited therapeutic benefits to varying degrees, with INPs@BL@Gel consistently showing the most pronounced improvements. Across multiple readouts, the INPs@BL@Gel group approached levels observed in healthy controls, suggesting a synergistic therapeutic effect among the components and further validating the feasibility of the proposed design strategy.

### 2.6 Colonic Barrier Restoration and Inflammation Resolution in DSS‐Induced Colitis

The loss of mucin‐producing goblet cells and disruption of the mucus barrier are key events in the onset and progression of UC, contributing significantly to disease exacerbation. To evaluate intestinal barrier integrity and the underlying mechanisms of barrier damage, intestinal tissue and blood samples were collected from the DSS, INPs, INPs@BL, INPs@BL@Gel, and healthy control groups.

**FIGURE 6:**
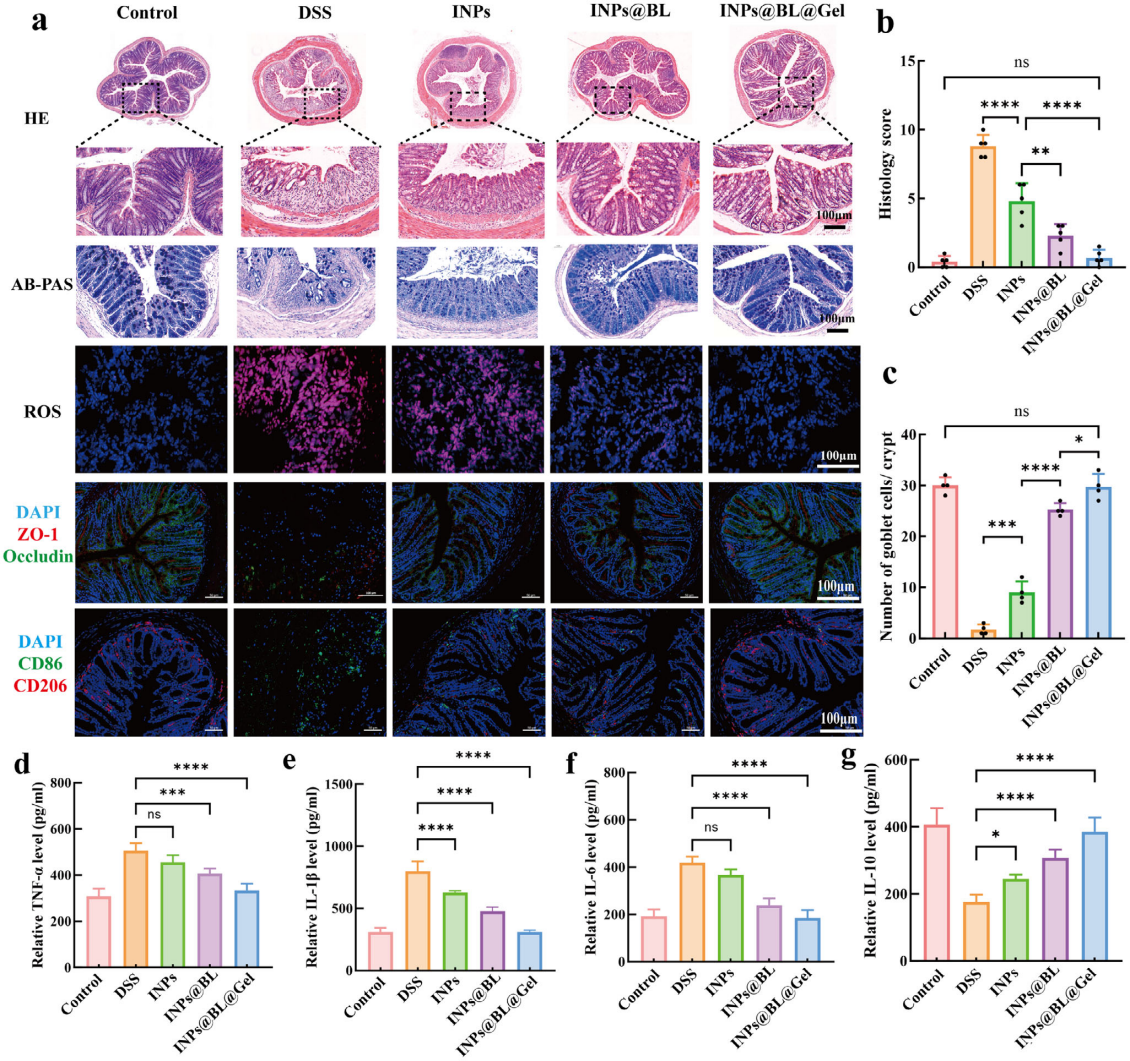
INPs@BL@Gel for the repair of colonic barrier damage and the resolution of inflammation. (a) Representative H&E, AB‐PAS, and ROS staining images of the colon tissue of mice treated with different drug groups; and the immunofluorescence co‐localization images of tight junction proteins (ZO‐1, Occludin) and macrophage markers (CD86, CD206) in the colon. (b) Histological injury scores of colon tissue after treatment with different drug groups. (c) The number of goblet cells in the colonic tissues of mice in each group. (d‐g) The levels of pro‐inflammatory cytokines (TNF‐α, IL‐1β, and IL‐6) and anti‐inflammatory cytokines (IL‐10) in the colon tissue of mice in each group were determined by ELISA. Data are expressed as Mean ± SD, with n = 5. Comparisons among multiple groups were performed using one‐way ANOVA, followed by Tukey's post hoc test for pairwise comparisons (^*^ *p* < 0.05, ^**^ *p* < 0.01, ^***^ *p* < 0.001, and ^****^ *p* < 0.0001).

Histological evaluation was first performed using hematoxylin and eosin (H&E) staining, and tissue damage was quantified using a comprehensive histological scoring system that accounts for inflammation severity, lesion depth, crypt damage, and the extent of affected areas (Table S4). As shown in Figure 6a, DSS‐induced colitis caused severe colonic injury, characterized by marked lymphocyte infiltration and crypt destruction, resulting in a histological score of approximately 9. In contrast, all treatment groups showed varying degrees of tissue repair, with the INPs@BL@Gel group exhibiting the most pronounced restoration of epithelial integrity, with the histological score reduced to approximately 1 (Figure 6b).

Subsequently, the ability of INPs@BL@Gel to repair the intestinal mucosal barrier was evaluated. Alcian blue‐periodic acid‐Schiff (AB‐PAS) staining demonstrated that INPs@BL@Gel effectively reversed inflammation‐induced damage to the mucus layer, restoring it toward a near‐normal state. This recovery was accompanied by a marked increase in goblet cell numbers and a thickening of the mucus layer (Figure 6a and Figure S15). Compared with the DSS‐induced colitis group, the number of goblet cells in the INPs@BL@Gel group increased approximately 20‐fold (Figure 6c). To quantify intestinal ROS levels, dihydroethidium (DHE) fluorescence imaging was performed. Among all treatment groups, the INPs@BL@Gel group exhibited the lowest fluorescence intensity, comparable to that of the healthy control group, indicating potent ROS‐scavenging capability (Figure 6a and Figure S16). Furthermore, INPs@BL@Gel significantly upregulated the expression of tight junction proteins ZO‐1 and occludin, further supporting restoration of intestinal barrier integrity (Figure 6a and Figure S17).

We also investigated the potential anti‐inflammatory effects of INPs@BL@Gel in DSS‐induced intestinal inflammation. Macrophages, as key immune effector cells, play a central role in regulating inflammatory responses; therefore, modulating macrophage polarization may help elucidate the therapeutic mechanism of INPs@BL@Gel in intestinal inflammation. Promoting the phenotypic transition from pro‐inflammatory M1 macrophages to anti‐inflammatory M2 macrophages is considered a promising strategy for restoring immune homeostasis in IBD. CD86 is a surface marker of M1 macrophages, whereas CD206 is widely used as a marker of M2 macrophages. Immunofluorescence staining of colon sections showed minimal CD86 expression in healthy mice, indicating immune homeostasis (Figure 6a and Figure S18). In contrast, DSS treatment resulted in a marked increase in CD86‐positive signals in colon tissue, suggesting a shift toward the pro‐inflammatory M1 phenotype. Conversely, treatment groups exhibited reduced CD86 expression accompanied by enhanced CD206 staining. Notably, the INPs@BL@Gel group showed almost undetectable CD86 signals, consistent with restoration of immune homeostasis and a tissue microenvironment favorable for mucosal repair.

To further validate the anti‐inflammatory mechanism, an enzyme‐linked immunosorbent assay (ELISA) was performed to quantify representative pro‐inflammatory and anti‐inflammatory cytokines in colonic tissue, including TNF‐α, IL‐1β, IL‐6 (pro‐inflammatory), and IL‐10 (anti‐inflammatory). As shown in Figure 6d–f, DSS exposure significantly increased the levels of TNF‐α, IL‐1β, and IL‐6 to 1.64‐, 2.57‐, and 2.18‐fold of those in healthy controls, respectively. In contrast, IL‐10 levels were reduced by 56.83% compared with healthy mice, indicating a pronounced inflammatory state in colitis (Figure 6g). Following treatment with INPs, INPs@BL, or INPs@BL@Gel, colonic inflammation was markedly alleviated, as evidenced by significant suppression of pro‐inflammatory cytokines and restoration of IL‐10 levels. Notably, compared with the DSS group, INPs@BL@Gel reduced TNF‐α, IL‐1β, and IL‐6 levels by 33.90%, 61.31%, and 55.82%, respectively, while increasing IL‐10 by 54.48%, outperforming both INPs and INPs@BL. These results are consistent with previous findings by the Yanling Hao group, which reported that *Bifidobacterium animalis subsp. lactis A6* reduced TNF‐α, IL‐1β, and IL‐6 levels by 48.6%, 45.5%, and 24.5%, respectively [42]. In comparison, the present probiotic‐based combinational strategy demonstrates a more pronounced anti‐inflammatory effect.

Collectively, these results demonstrate that INPs@BL@Gel not only exerts significant therapeutic effects in DSS‐induced colitis but also promotes intestinal barrier repair and inflammation resolution. It achieves the most pronounced mucosal healing effect among all treatment groups, while also effectively modulating immune responses and suppressing inflammatory activity.

### 2.7 Alleviation of Colitis‐Associated Depression

**FIGURE 7:**
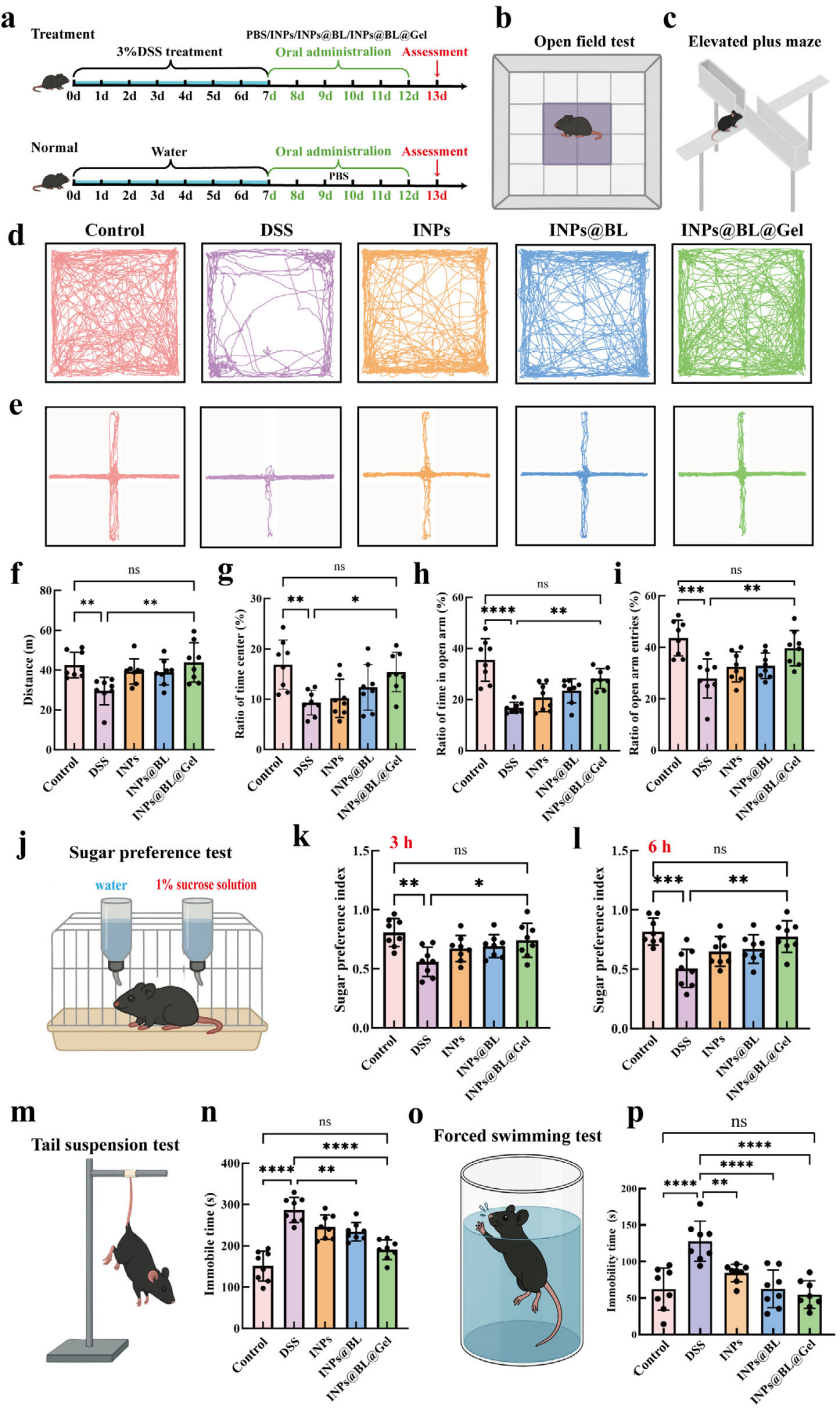
Behavioral Analysis of INPs@BL@Gel for Treating colitis‐associated depression. (a) Schematic diagram of the modeling and treatment process. (b, c) OFT schematic diagram and EPM schematic diagram. (d) Representative images of motion trajectory tracking of mice treated with different drug groups in OFT. (e) Representative images of the movement trajectory tracking of mice in each group in the EPM. (f) Total movement distance of mice in each group of OFT. (g) Proportion of central time of mice in each group of OFT. (h) The proportion of time that mice in each group entered the open arms in EPM. (i) The proportion of the number of times mice in each group entered the open arm in the EPM. (j) Schematic diagram of SPT. (k, l) The sugar water preference rates of mice in each group at 3 and 6 h in SPT. (m, n) TST schematic diagram and the immobility time of each group. (o, p) FST schematic diagram and the immobility time of each group. Data are expressed as Mean ± SD, with n = 8. Comparisons among multiple groups were performed using one‐way ANOVA, followed by Tukey's post hoc test for pairwise comparisons (^*^ *p* < 0.05, ^**^ *p* < 0.01, ^***^ *p* < 0.001, and ^****^ *p* < 0.0001).

DSS‐induced colitis mice often exhibit significant anxiety‐ and depression‐like behaviors, such as reduced locomotor activity and impaired exploratory behavior. This section evaluates whether oral administration of INPs@BL@Gel can alleviate colitis‐associated anxiety‐ and depression‐like phenotypes using five behavioral paradigms: the open field test (OFT), elevated plus maze (EPM), sucrose preference test (SPT), TST, and forced swimming test (FST). Mice were administered 3% DSS for 7 days to induce colitis, followed by oral treatment with PBS, INPs, INPs@BL, or INPs@BL@Gel for 5 consecutive days. Behavioral assessments were then conducted to evaluate anxiety‐ and depression‐like behaviors (Figure 7a).

In the OFT, DSS‐treated mice exhibited stereotyped movement patterns, rarely entering the central area and traveling shorter distances (Figure 7b,d). Quantitative analysis showed that the DSS group displayed nearly one‐third reduction in total movement distance and a 50% decrease in time spent in the central area compared with healthy controls (Figure 7f,g). After treatment, mice in the INPs, INPs@BL, and INPs@BL@Gel groups showed improved exploratory behavior and reduced repetitive locomotion patterns. Notably, the INPs@BL@Gel group exhibited the most pronounced improvement, with a total distance of approximately 43.83 m and a central zone occupancy of 15.45%, approaching levels observed in healthy controls, indicating substantial alleviation of anxiety‐ and depression‐like behaviors (Figure 7d).

In the EPM test, DSS‐treated mice showed reduced time spent in the open arms and fewer open arm entries, indicating increased anxiety‐like behavior. Mice treated with INPs@BL@Gel exhibited the strongest anxiolytic effect, with a 1.68‐fold increase in open arm residence time and a 1.35‐fold increase in open arm entries compared with the DSS group (Figure 7c,e,h,i).

In the SPT, sucrose consumption during the first 3 h reflected the immediate reward response to initial exposure. As shown in Figure 7j–l, the DSS group showed approximately one‐third lower sucrose preference than the healthy control group, indicating reduced responsiveness to rewarding stimuli. At 6 h, sucrose preference further declined by approximately two‐fifths, suggesting persistent anhedonia‐like behavior. Notably, INPs@BL@Gel treatment largely restored sucrose preference, with indices of 0.74 and 0.78 at 3 h and 6 h, respectively, approaching those of healthy mice. These results indicate that DSS‐induced mice developed a pronounced anhedonia‐like phenotype, a core feature of depression, which was significantly ameliorated by all treatment groups, particularly INPs@BL@Gel.

In the TST and FST, DSS‐induced mice exhibited significantly prolonged immobility times, with TST and FST immobility increasing to approximately 1.93‐fold and 2.17‐fold of healthy controls, respectively. Notably, INPs@BL@Gel treatment reduced immobility by 37.9% in the TST and 62.96% in the FST, demonstrating a more pronounced effect than the other treatment groups (Figure 7m–p). A previous study by the Honghong Yao group reported an approximately one‐third reduction in immobility time in NLRP3‐deficient mice following microbiota‐based intervention [43]. In comparison, our INPs@BL@Gel group achieved reductions of 37.9% and 62.96% in the TST and FST, respectively, highlighting its strong therapeutic efficacy in alleviating anxiety‐ and depression‐like behaviors.

Taken together, these behavioral assessments consistently demonstrate that DSS exposure induces anxiety‐ and depression‐like behaviors in mice, while INPs@BL@Gel markedly ameliorates these colitis‐associated depressive phenotypes. These findings further support the role of the gut‐brain axis in mediating the therapeutic effects of INPs@BL@Gel in colitis‐associated depression.

### 2.8 Restoration of BBB Integrity and Neural Function

**FIGURE 8:**
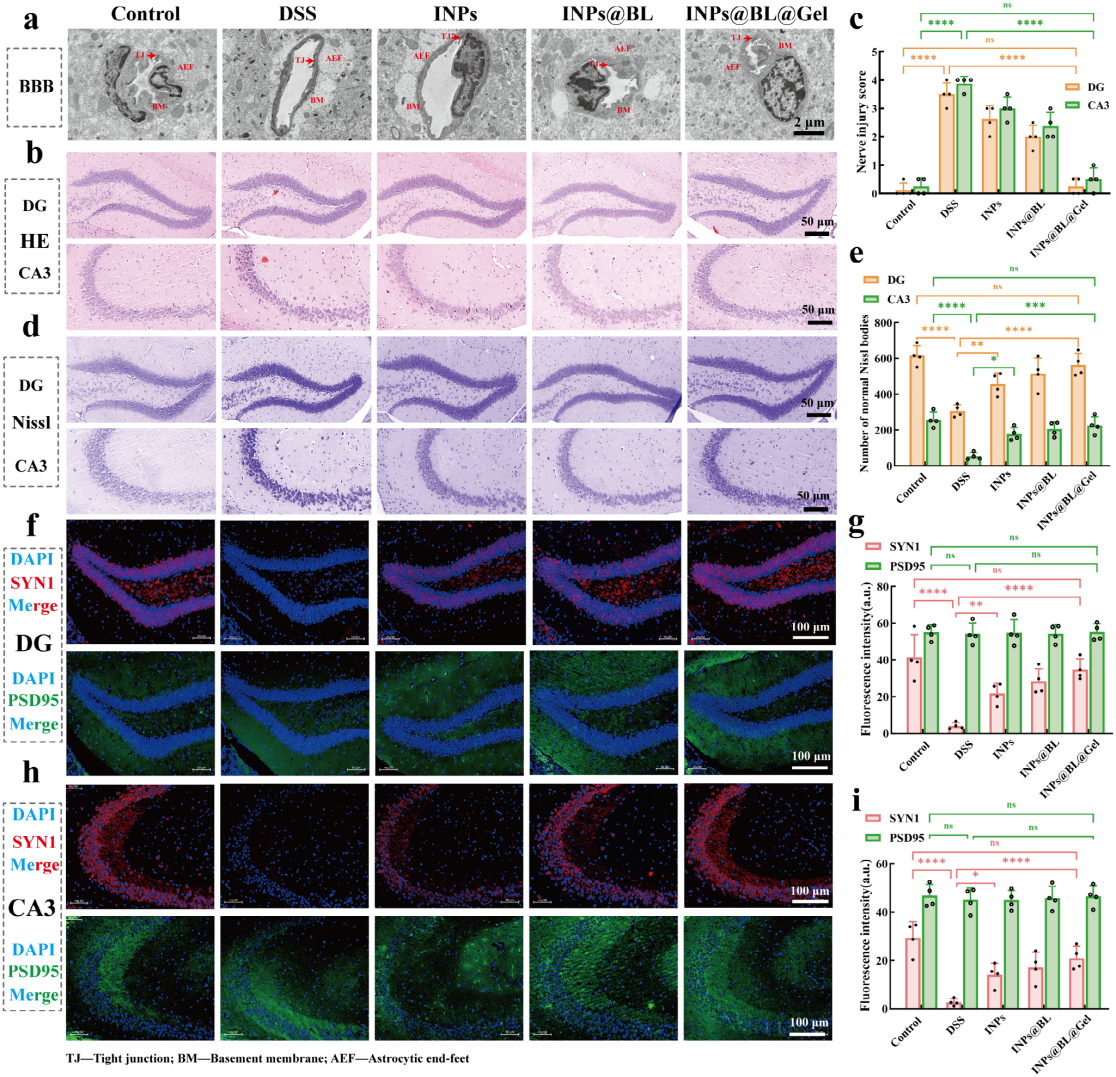
INPs@BL@Gel promotes neuronal repair and synaptic plasticity. (a) Representative TEM images of the BBB in mice after treatment with different drug groups. (b) Representative H&E staining images and neuronal injury scores of the DG and CA3 regions in the hippocampus of mice after treatment with different drug groups (c). (d) Representative Nissl staining of active neurons in the DG and CA3 regions of the hippocampus in each group of mice and the number of intact Nissl bodies (e). (f) Representative immunofluorescence co‐localization images and quantitative analysis of synapse‐associated proteins (SYN1 and PSD95) in the DG region of the hippocampus of mice in each group (g). (h) Representative immunofluorescence co‐localization images and quantitative analysis of synaptic‐associated proteins (SYN1 and PSD95) in the hippocampal CA3 region of mice in each group (i). Data are expressed as Mean ± SD, with n = 4. Comparisons among multiple groups were performed using one‐way ANOVA, followed by Tukey's post hoc test for pairwise comparisons (^*^ *p* < 0.05, ^**^ *p* < 0.01, ^***^ *p* < 0.001, and ^****^ *p* < 0.0001).

Based on the behavioral outcomes, we hypothesized that DSS‐induced colitis‐associated depression may result from the leakage of gut lumen‐derived molecules (GLDMs) and toxins from inflamed colonic tissue into the circulation. These circulating factors may initially compromise BBB integrity, subsequently triggering neuroinflammation and local immune responses in the brain. To test this hypothesis, BBB integrity was evaluated using TEM and Evans Blue (EB) extravasation assays. As shown in Figure 8a, TEM images revealed pronounced ultrastructural abnormalities in the BBB of DSS‐induced mice, including basement membrane disruption, tight junction disassembly, and astrocytic swelling. EB staining further confirmed increased dye accumulation in the brain, indicating elevated BBB permeability (Figure S19). In contrast, oral administration of INPs@BL@Gel effectively reversed these pathological alterations and preserved BBB structural integrity.

H&E staining and Nissl staining further revealed significant damage in the hippocampal region of DSS‐induced mice (Figure 8b,d). The dentate gyrus (DG) and CA3 regions exhibited reduced neuronal survival, marked neuronal degeneration, decreased Nissl body density, and overall lower neuronal density compared with healthy controls. In contrast, no obvious pathological alterations were observed in the DG or CA3 regions of mice treated with INPs@BL@Gel. Quantitative analysis of H&E and Nissl staining results further supported these observations (Figure 8c,e and Table S5). The histological injury scores in the DSS group were approximately 3.5 in the DG and 3.875 in the CA3 region. Following INPs@BL@Gel treatment, these scores decreased to 0.25 (DG) and 0.5 (CA3), approaching those of healthy controls. Moreover, the number of intact Nissl bodies in the DG and CA3 regions of DSS‐treated mice decreased to approximately one‐half and one‐fifth of those in healthy controls, respectively, whereas INPs@BL@Gel treatment increased these values by 1.837‐fold (DG) and 4.28‐fold (CA3) compared with the DSS group. These findings indicate that INPs@BL@Gel effectively reverses DSS‐induced hippocampal neuronal damage and structural abnormalities.

To further assess synaptic plasticity, immunofluorescence staining was performed to evaluate the expression of the presynaptic protein synapsin‐1 (SYN1) and the postsynaptic density protein PSD95 in the hippocampal DG and CA3 regions. Changes in these presynaptic and postsynaptic markers provide insights into synaptic remodeling. As shown in Figure 8f,h, DSS exposure markedly reduced SYN1 expression in both the DG and CA3 regions. Quantitative fluorescence analysis revealed that SYN1 expression in the CA3 region decreased by 90.65% in the DSS group compared with controls. Relative to the DSS group, SYN1 fluorescence increased by 5.14‐fold (INPs), 6.62‐fold (INPs@BL), and 7.64‐fold (INPs@BL@Gel) (Figure 8i and Figure S20). A similar trend was observed in the DG region, where SYN1 fluorescence in the DSS group decreased by 98.39% compared with controls, while INPs, INPs@BL, and INPs@BL@Gel treatments increased SYN1 expression by 5.36‐fold, 6.95‐fold, and 8.59‐fold, respectively (Figure 8g and Figure S21). Notably, PSD95 staining showed relatively weak expression across all groups, with no significant differences in fluorescence intensity. Additionally, high‐performance liquid chromatography (HPLC) analysis demonstrated that HVA could be generated only in the presence of both BL and Tyr, whereas no HVA production was detected when Tyr or BL was incubated alone in the medium (Figure S22). These findings suggest that INPs can be metabolized by BL to produce HVA. Collectively, these results indicate that BL may improve colitis‐associated behavioral abnormalities partly by preserving presynaptic integrity in the hippocampus and alleviating synaptic dysfunction associated with anxiety‐ and depression‐like states through a Tyr/HVA‐associated pathway.

In summary, our findings provide mechanistic insights into DSS‐induced colitis‐associated depression and demonstrate that INPs@BL@Gel represents an effective strategy for preserving BBB integrity, improving synaptic plasticity, and potentially restoring neural function.

### 2.9 Metagenomic Analysis

**FIGURE 9:**
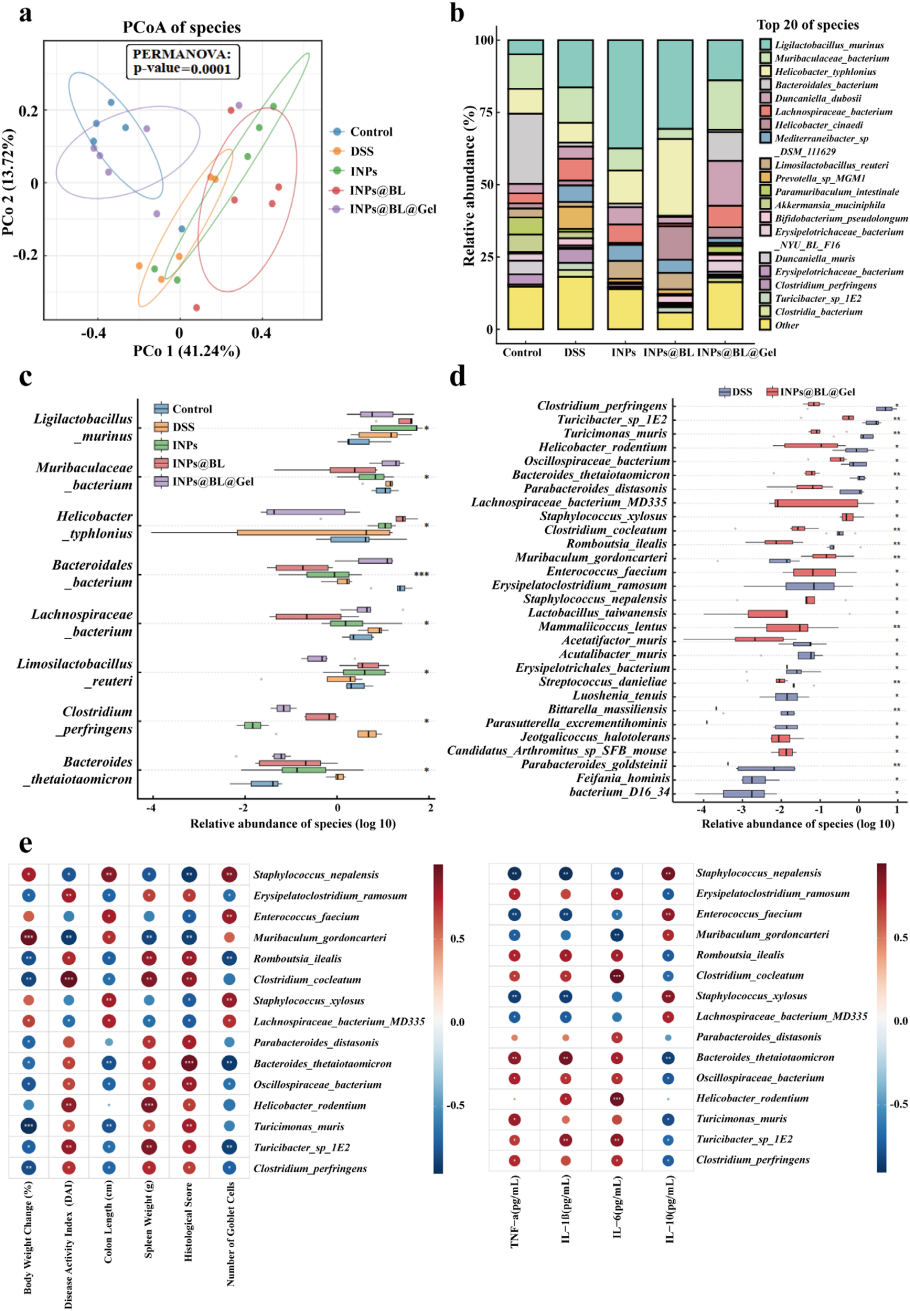
Regulation of INPs@BL@Gel on the homeostasis of intestinal microbiome. (a) The beta diversity of the intestinal microbiota of mice in different drug treatment groups is shown through PCoA (PERMANOVA test). (b) The relative abundance of the Top 20 differentially abundant species in the intestinal microbiota of each group of samples. (c) The relative abundance of bacteria with representative differences in each group of intestines (Kruskal‐Wallis test). (d) Relative abundance of intestinal representative differential bacteria in the DSS group and INPs@BL@Gel group (Kruskal‐Wallis test). (e) Spearman correlation analysis between differential bacteria and inflammation‐related phenotypes and indicators. n = 5. ^*^ *p* < 0.05, ^**^ *p* < 0.01, ^***^ *p* < 0.001 and ^****^ *p* < 0.0001.

The gut microbiota plays a critical role in the pathogenesis of colitis‐associated depression. Dysbiosis in this context is typically characterized by reduced microbial diversity and the expansion of pathogenic or opportunistic taxa. To investigate the impact of the engineered INPs@BL@Gel on gut microbial composition, metagenomic sequencing was performed. Principal coordinates analysis (PCoA) based on species‐level abundance profiles revealed clear differences in microbial community structure among groups (Figure 9a). The DSS‐treated group was distinctly separated from the healthy control group, indicating severe colitis‐induced dysbiosis. In contrast, INPs@BL@Gel treatment markedly reversed this dysregulation, with samples clustering closer to those of the control group in PCoA space, suggesting substantial recovery of the gut microbiota toward a homeostatic state. Phylogenetic analysis (Figure 9b), together with Kruskal‐Wallis testing (Figure 9c), identified eight discriminant species that most strongly contributed to the observed intergroup differences, supporting partial yet targeted restoration of the microbial ecosystem following INPs@BL@Gel administration.

Although BL was not among the taxa showing the most pronounced intergroup differences, quantitative analysis revealed that BL abundance was significantly increased only in the INPs@BL@Gel group (Figure S23). This finding suggests that the hydrogel‐based formulation effectively enhances the survival and intestinal colonization of the administered probiotic strain. Pairwise comparison between the DSS and INPs@BL@Gel groups further demonstrated selective suppression of inflammation‐associated pathogenic taxa (Figure 9d). DSS‐induced colitis resulted in a marked enrichment of *Clostridium perfringens* and *Helicobacter rodentium*, both of which are known to exacerbate intestinal inflammation through toxin production and immune activation. In addition, a significant increase in *Bacteroides thetaiotaomicron* was observed in the DSS group. Although generally considered a commensal organism under physiological conditions, this species can adopt a mucin‐degrading phenotype during inflammatory states, thereby compromising mucus barrier integrity. Notably, INPs@BL@Gel treatment reduced the abundance of these taxa to levels comparable with those in healthy controls, consistent with restoration of epithelial integrity and mucus barrier function, which likely limits the ecological niches available for opportunistic pathobionts.

Concurrently, INPs@BL@Gel restored bacterial taxa that were depleted in DSS‐induced colitis, including *Muribaculum gordoncarteri* and *Lachnospiraceae bacterium* MD335, both of which are recognized SCFA‐producing bacteria that support colonic energy metabolism and epithelial integrity. Spearman correlation analysis further linked these microbiota shifts to host phenotypic and inflammatory parameters (Figure 9e). DSS‐enriched taxa, such as *C. perfringens* and *B. thetaiotaomicron*, showed positive correlations with disease severity indicators (DAI score, histological score, and spleen weight) as well as pro‐inflammatory cytokines (IL‐1β, IL‐6, and TNF‐α), while showing negative correlations with body weight recovery and colon length. In contrast, taxa restored by INPs@BL@Gel, including *Muribaculum gordoncarteri* and *Lachnospiraceae bacterium* MD335, exhibited the opposite correlation pattern and were positively associated with the anti‐inflammatory cytokine IL‐10. Further comparative analysis of pathway abundance (Figure S24) revealed several key metabolic pathways. Although the relative abundance of these pathways at the genomic (DNA) level did not differ significantly among groups (*p* > 0.05), the overall functional profile of the INPs@BL@Gel group showed a clear restoration trend. This suggests that the therapeutic effects are likely driven by the selective expansion of bacterial taxa carrying these functional capacities, such as members of the *Lachnospiraceae* family, thereby contributing to the metabolic alterations observed in subsequent metabolomic analyses.

Overall, the metagenomic data demonstrate that INPs@BL@Gel induces targeted microbiome remodeling, characterized by suppression of mucin‐associated pathobionts and enrichment of SCFA‐producing commensal bacteria. These microbiota changes are closely associated with improvements in intestinal inflammation and colitis‐associated neurobehavioral symptoms, supporting a microbiota‐mediated mechanism underlying the therapeutic efficacy of INPs@BL@Gel.

### 2.10 Targeted Metabolite Analysis

**FIGURE 10:**
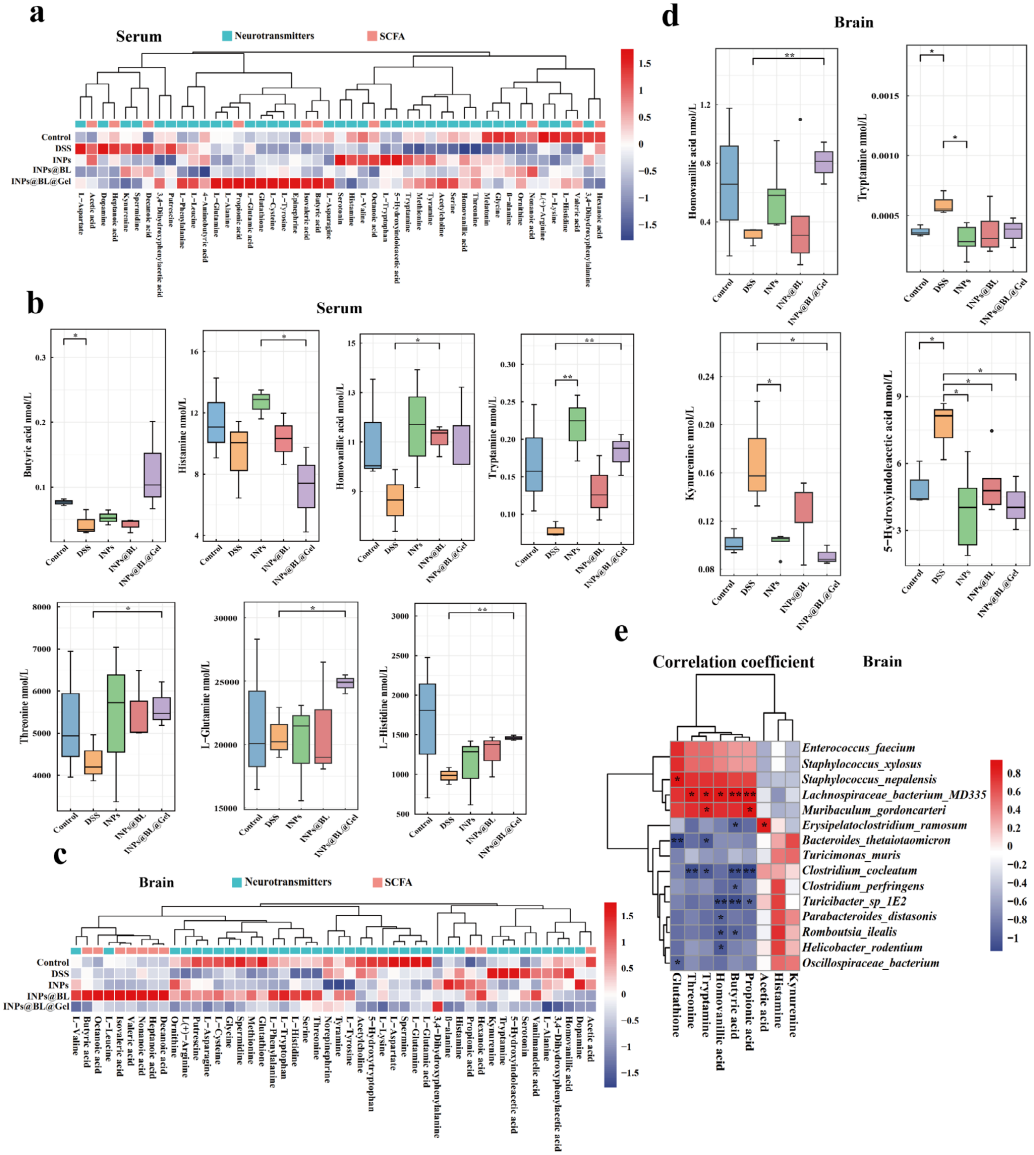
Targeted metabolomics analysis of serum and brain tissue after INPs@BL@Gel treatment. (a) Hierarchical clustering analysis of neurotransmitters and SCFAs in mouse serum following treatment with different drug groups, along with quantitative analysis of differential neurotransmitters and SCFAs (b). (c) Hierarchical clustering analysis of differential neurotransmitters and SCFAs in brain tissue across groups, and quantitative analysis of differential neurotransmitters and SCFAs (d). (e) Spearman correlation analysis between representative gut microbiota and levels of differential neurotransmitters and SCFAs in brain tissue. Data are expressed as Mean ± SD, with n = 4.

To evaluate the functional consequences of microbiota remodeling, targeted metabolomic analyses were performed in both serum and brain tissues (for detailed methods, see Supporting Information). Hierarchical clustering of serum metabolites revealed marked metabolic dysregulation in DSS‐treated mice (Figure 10a). Several metabolites, including HVA, butyrate, threonine, and histidine, were significantly reduced following DSS exposure. These alterations were largely reversed after INPs@BL@Gel treatment. Quantitative analyses further confirmed the partial restoration of microbially derived metabolites and essential nutrients in circulation (Figure 10b). Among the SCFAs, serum butyrate levels were significantly decreased in DSS‐treated mice (*p* < 0.05). Treatment with INPs@BL@Gel produced a clear increasing trend in butyrate abundance. Although the difference did not reach statistical significance because of inter‐individual variability, the mean levels approached those of healthy controls. Previous studies have shown that butyrate contributes to BBB repair and endothelial homeostasis, supporting its potential role in the neuroprotective effects observed in this study.

We next examined metabolites associated with neurotransmitter pathways. Serum HVA, the primary metabolite of dopamine, was significantly increased in the INPs@BL@Gel group compared with the DSS group (*p* < 0.05), suggesting partial restoration of peripheral dopamine metabolism. In addition, serum tryptamine, a microbiota‐derived neuromodulator capable of crossing the BBB, was markedly depleted after DSS treatment but significantly restored following INPs@BL@Gel administration (*p* < 0.01). Consistent with these peripheral alterations, brain metabolomic profiling demonstrated corresponding improvements in central neurochemical homeostasis. Heatmap analysis revealed recovery trends in several neuroactive precursors and in the antioxidant glutathione (Figure 10c). Quantitative analysis further showed that kynurenine levels were significantly elevated in the brains of DSS‐treated mice (*p* < 0.05), indicating activation of the indoleamine 2,3‐dioxygenase (IDO) pathway (Figure 10d). INPs@BL@Gel treatment significantly reduced kynurenine levels (*p* < 0.05), thereby attenuating this neurotoxic metabolic pathway. Moreover, 5‐hydroxyindoleacetic acid (5‐HIAA), the major serotonin metabolite, was abnormally elevated in DSS‐treated mice and significantly normalized after treatment (*p* < 0.05), indicating restoration of disrupted serotonin metabolism.

To further associate metabolic alterations with microbiota remodeling, Spearman correlation analysis was performed. *Lachnospiraceae bacterium MD335* and *Muribaculum gordoncarteri*, both enriched after INPs@BL@Gel treatment, showed positive correlations with serum tryptamine and valeric acid levels (Figure 10e). The simultaneous recovery of butyrate and tryptophan‐derived metabolites provides a plausible mechanistic basis for BBB reinforcement. Butyrate, a potent histone deacetylase (HDAC) inhibitor, has been reported to enhance tight junction protein expression in brain endothelial cells through epigenetic regulation [44, 45]. Tryptamine, acting as a ligand for the aryl hydrocarbon receptor (AhR), contributes to vascular endothelial integrity [46, 47]. Together, these signaling pathways may reduce BBB permeability, limit infiltration of peripheral pro‐inflammatory cytokines, and suppress diversion of tryptophan toward the kynurenine pathway, thereby alleviating central neurochemical imbalance. Notably, HVA levels increased in both serum and brain tissues following INPs@BL@Gel treatment. HVA has been associated with modulation of synaptic plasticity, particularly within the prefrontal cortex and hippocampal circuits involved in learning, emotional regulation, and cognitive recovery [21]. Improved dopamine metabolism may therefore further support synaptic stability and remodeling by reducing oxidative stress and improving the neuroinflammatory microenvironment.

Collectively, the INPs@BL@Gel composite system exhibited sustained and comprehensive therapeutic efficacy against DSS‐induced colitis and its associated neuropsychiatric comorbidities. These effects were supported by coordinated improvements in intestinal inflammation, behavioral phenotypes, barrier integrity, and gut microbiota homeostasis. In the DSS colitis model, INPs@BL@Gel restored colon length to near‐normal levels, reaching approximately 1.51‐fold that of the DSS group and fully reversing the 33.04% shortening induced by DSS. The treatment also increased goblet cell abundance in colonic tissue by 20.3‐fold, indicating effective repair of mucus barrier damage. Intestinal inflammation was markedly suppressed. Levels of the pro‐inflammatory cytokines TNF‐α, IL‐1β, and IL‐6 decreased by 33.9%, 61.31%, and 55.82%, respectively, whereas the anti‐inflammatory cytokine IL‐10 increased by 54.48%. Consistent with these molecular findings, histopathological injury scores decreased from 9 in DSS‐treated mice to 1 after treatment, approaching healthy control levels.

Behavioral assessments further demonstrated near‐complete normalization of colitis‐associated depressive‐ and anxiety‐like behaviors. In the open‐field test, treated mice traveled 43.83 m and spent 15.45% of their time in the central zone, effectively reversing the approximately one‐third reduction in locomotor activity and the nearly 50% decrease in center exploration induced by DSS. In the elevated plus maze, the proportion of time spent in the open arms and the number of open‐arm entries increased by 1.68‐fold and 1.35‐fold, respectively, compared with DSS‐treated mice. In the sucrose preference test, preference indices reached 0.74 at 3 h and 0.78 at 6 h, whereas DSS treatment reduced these values by approximately one‐third and two‐fifths, respectively. Furthermore, immobility times in the tail suspension and forced swim tests decreased by 37.9% and 62.96%, respectively. No significant differences were observed between treated mice and healthy controls across the behavioral paradigms.

Mechanistically, INPs@BL@Gel restored intestinal barrier integrity by upregulating ZO‐1 and occludin expression, eliminating excessive ROS (91.0% clearance), and rescuing epithelial viability after H_2_O_2_ challenge from 74.5% to >90%. Concurrently, BBB integrity was re‐established, as evidenced by normalized EB leakage, increased numbers of morphologically intact Nissl bodies in the hippocampal DG and CA3 regions (1.84‐fold and 4.28‐fold versus DSS‐treated mice), and enhanced SYN1 expression (7.64‐fold and 8.59‐fold, respectively). At the microbiota level, INPs@BL@Gel suppressed inflammation‐associated taxa, including *Clostridium perfringens* and *Helicobacter rodentium*, while enriching SCFA‐producing commensals such as *Muribaculum gordoncarteri* and *Lachnospiraceae bacterium MD335*. Principal coordinate analysis (PCoA) further demonstrated substantial convergence toward the healthy microbial profile, collectively indicating restoration of gut‐brain axis homeostasis.

The overall therapeutic efficacy of INPs@BL@Gel arises from a gut‐brain dual‐site intervention strategy delivered through two complementary mechanisms: (I) targeted suppression of intestinal inflammation through a cascade involving “site‐specific release at inflamed regions‐multicomponent synergy across pathological targets‐microbiota remodeling”; and (II) alleviation of neuropsychiatric symptoms through “complementary component functions‐directed metabolite regulation‐multitarget intervention”. INPs@BL@Gel is a composite hydrogel system centered on BL and rationally integrated with Bai, Inu, and Tyr to simultaneously address intestinal and neurological dysfunction. Following oral administration, INPs@BL@Gel resists gastrointestinal degradation, thereby enabling prolonged intestinal retention, stable bacterial survival, and effective colonization. Within inflamed intestinal regions, Bai, Inu, Tyr, and BL are locally released and synergistically target multiple pathological processes to alleviate IBD while reshaping the gut microbiota. In parallel, the system modulates intestinal metabolite profiles by increasing neuroactive metabolites, including HVA, tryptamine, and SCFAs such as butyrate and valerate. These changes may contribute to multitarget regulation of anxiety‐ and depression‐like behaviors while reducing kynurenine and abnormally elevated 5‐HIAA. Through gut‐brain axis signaling, these coordinated metabolic alterations may further promote BBB repair and improve neuropsychiatric symptoms. Collectively, each component of INPs@BL@Gel performs multiple complementary functions, resulting in enhanced overall therapeutic efficacy. This design concept and construction strategy provide experimental support for engineered probiotic systems and gut‐brain dual‐site synergistic therapies.

## 3 Conclusions

In response to the clinical challenge posed by the frequent comorbidity of inflammatory bowel diseases (IBDs) with neuropsychiatric disorders, such as depression and anxiety, this study proposes a “gut‐brain dual‐site, multi‐target, integrated synergistic, and metabolite‐regulated co‐therapeutic strategy.” This strategy was specifically designed to overcome the limitations of conventional oral pharmacotherapy, which is restricted by the spatial separation between intestinal and cerebral therapeutic targets, as well as the complex bidirectional regulation mediated through the gut‐brain axis. To implement this concept, the INPs@BL@Gel composite system was rationally constructed. Bai (anti‐inflammatory and neuroprotective agent), Tyr (precursor of HVA), Inu (prebiotic and promoter of SCFA production), and BL were integrated into a coordinated polymeric network through Fe(III)‐mediated coordination to form INPs. Encapsulation within a dual‐responsive hydrogel enabled inflammation‐triggered release at colonic lesions. This design established a stepwise therapeutic cascade involving multicomponent synergy, metabolic profile modulation, and gut‐brain axis co‐regulation. The system not only restored beneficial metabolites, including normalization of HVA levels and elevation of butyrate toward levels observed in healthy controls, but also replenished essential amino acids, such as threonine and glutamine, that are required for intestinal mucosal repair. Collectively, this work introduces a new paradigm in probiotic engineering characterized by on‐demand integration of functional components and controllable optimization of metabolic outcomes.

By integrating Bai and BL through a multitarget coordination strategy, INPs@BL@Gel exploited the pH/MMP dual‐responsive and acid‐resistant properties of SG‐Gel to effectively protect probiotic viability. As a result, BL survival under gastric acid conditions increased by 56.87‐fold, while intestinal colonization increased by 10.58‐fold. In addition, colon length was restored by 1.51‐fold, and goblet cell abundance increased by 20.3‐fold, collectively demonstrating robust therapeutic efficacy in IBD treatment. Importantly, through regulation of key gut‐derived metabolites, including HVA, SCFAs (such as butyrate and valerate), and amino acids (e.g., threonine and histidine), the system may also markedly alleviate colitis‐associated behavioral abnormalities. INPs@BL@Gel restored SYN1 expression in the hippocampal DG region to levels 8.59‐fold higher than those observed in DSS‐treated mice, reduced neurotoxic metabolites such as kynurenine, and reversed associated neuropathological alterations. Behavioral evaluations, including the open‐field and tail suspension tests, together with neuromolecular analyses of SYN1 expression and Nissl body quantification, demonstrated that depression‐like phenotypes in treated mice were largely indistinguishable from those of healthy controls. Furthermore, INPs@BL@Gel exhibited pronounced bidirectional barrier‐protective effects by simultaneously preserving intestinal epithelial integrity and restoring BBB function. These findings provide experimental support for the pathophysiological concept of “gut‐derived mental disorders” and highlight the efficacy of INPs@BL@Gel in suppressing intestinal inflammation, alleviating neuropsychiatric symptoms, restoring barrier integrity, and re‐establishing gut microecological homeostasis. Overall, this study presents a promising oral and long‐acting co‐therapeutic strategy for the treatment of colitis and its associated depression, thereby providing a foundation for future large‐animal studies and potential clinical translation.

## 4 Experimental Section

### 4.1 Materials and Strains

Bai, ferric chloride (FeCl_3_), gelatin, genipin, calcium chloride (CaCl_2_), and N,N‐dimethylformamide (DMF) were purchased from Aladdin (Shanghai, China). Tyr, Inu, and polyethylene glycol (PEG 20000) were obtained from Macklin Biochemical Co., Ltd. (Shanghai, China). Dextran sulfate sodium (DSS; MW 36–50 kDa) was purchased from MP Biomedicals (California, USA). *Bifidobacterium longum ATCC BAA‐999* was obtained from Ningbo Junyou Medical Technology Development Co., Ltd. Silkworm cocoons were provided by Jiangsu Fu'an Cocoon Silk Co., Ltd. (Yancheng, China). TPY liquid medium was purchased from Haibo Biotechnology Co., Ltd. (Qingdao, China). 2′,7′‐dichlorodihydrofluorescein diacetate (DCFH‐DA) was obtained from Kaiji Biotechnology Co., Ltd. (Nanjing, China). Antibodies for immunofluorescence staining, including PSD95 (RRID: AB_3102405), SYN1 (RRID: AB_10952778), occludin (RRID: AB_3070241), and ZO‐1 (RRID: AB_3675805), were purchased from Hangzhou Hua'an Biotechnology Co., Ltd. (Hangzhou, China). All reagents were of analytical grade and used as received unless otherwise specified.

### 4.2 Preparation of ICPs and INPs

DMF solutions of Bai (5 mg mL^−1^) and FeCl_3_ (5 mg mL^−1^), as well as aqueous solutions of Tyr (0.45 mg mL^−1^) and Inu (5 mg mL^−1^), were prepared in advance. To synthesize the coordination polymer nanoparticles, 0.1 mL of the Bai solution, 0.2 mL of the Tyr solution, and 0.2 mL of the FeCl_3_ solution were sequentially added to 10 mM Tris buffer containing Pluronic F127 (0.4 wt.%). The mixture was stirred at room temperature for 1 h to allow coordination self‐assembly, yielding Bai‐Fe(III)‐Tyr coordination polymer nanoparticles (ICPs). Subsequently, 0.2 mL of the Inu solution was added to the ICP dispersion and stirred continuously for an additional 4 h to form Inu‐coated Bai‐Fe(III)‐Tyr nanoparticles (INPs). The resulting nanoparticles were purified by dialysis (3.5 kDa MWCO dialysis bag), followed by centrifugal ultrafiltration (3 kDa filters, 2000 rpm, 15 min) to remove unreacted components and residual solvents.

### 4.3 Preparation of Silk Fibroin‐Gelatin Composite Hydrogel (SG‐Gel)

Silkworm cocoons were cut into small pieces and boiled in 0.5 wt.% sodium carbonate solution to remove sericin, followed by thorough rinsing with deionized water and drying. The degummed SF (20 g) was dissolved in a ternary solvent system composed of CaCl_2_, ethanol, and deionized water (molar ratio 1:2:8) under heating and stirring until complete dissolution. The solution was centrifuged at 4500 rpm for 10 min, filtered, and dialyzed against deionized water to obtain a 2 wt.% SF solution. The SF solution was then concentrated osmotically against 20 wt.% PEG 20000 solution for 24 h to yield a 5 wt.% SF solution.

Separately, a 5 wt.% gelatin solution was prepared, and 1 wt.% genipin solution was added as a crosslinker with thorough mixing. The concentrated SF solution was subsequently incorporated into the gelatin mixture, followed by incubation at 37°C for 4 h to form the SG‐Gel.

### 4.4 Preparation of INPs@BL@Gel

BL was revived and cultured in TPY liquid medium to the stationary phase. One milliliter of bacterial suspension was collected, centrifuged, and washed twice with sterile PBS. Subsequently, 200 µL of INPs solution (50 mg mL^−1^) was added to 1 mL of the BL suspension and gently mixed at room temperature for 1 h to allow nanoparticle association, yielding INPs@BL. The product was collected by centrifugation and washed twice with PBS to remove unbound nanoparticles. Finally, the INPs@BL suspension was resuspended in the SG‐Gel precursor mixture and incubated at 37°C for 4 h to obtain the composite formulation INPs@BL@Gel. The parameter optimization and determination of optimal conditions for the INPs@BL and SG‐Gel carrier systems are shown in Table S1.

### 4.5 Characterization of INPs@BL@Gel

The hydrodynamic diameter and zeta potential of INPs were measured using a Malvern particle size analyzer (Zetasizer Nano ZS90, Malvern Instruments Ltd., UK). The morphology of the nanoparticles was examined by high‐resolution transmission electron microscopy (HRTEM, JEM‐F200, JEOL, Japan). HAADF‐STEM (JEM‐ARM200F, JEOL, Japan) was used to analyze the elemental distribution and composition of INPs@BL. The microstructures of SG‐Gel and INPs@BL@Gel were observed by SEM (Sigma 360, Carl Zeiss, Germany). UV–vis spectroscopy (T60, PG Instruments, China) and Fourier transform infrared (FTIR, TENSOR 27, Bruker, Germany) spectroscopy were used to characterize the chemical composition of INPs and INPs@BL. HPLC (1260 Infinity II, Agilent Technologies, USA) was used to quantify the loading content and relative ratios of active components in the nanoparticles. Chromatographic separation was performed using acetonitrile as mobile phase A and 0.5% phosphoric acid aqueous solution as mobile phase B under gradient elution conditions. (XPS, ESCALAB Xi^+^, Thermo Fisher Scientific, USA) was used to analyze the surface chemical composition of INPs. (XRD, D8 ADVANCE, Bruker, Germany) was conducted to evaluate their crystalline structure. Fluorescence images were acquired using a super‐resolution laser scanning confocal microscope (TCS SP8 STED 3X, Leica Microsystems, Germany) and a whole‐slide digital pathology system (Axioscan 7, Carl Zeiss Microscopy, Germany).

### 4.6 Cell Lines and Culture Conditions

Mouse hippocampal neuronal cells (HT22, RRID: CVCL_0321) and human normal colon epithelial cells (NCM460, RRID: CVCL_0460) were obtained from ProNova Biotechnology Co., Ltd. (Wuhan, China). Both HT22 and NCM460 cells were cultured in Dulbecco's Modified Eagle Medium (DMEM) supplemented with 10% fetal bovine serum. All cells were maintained at 37°C in a humidified incubator with 5% CO_2_.

### 4.7 External Environmental Resistance and Responsive Release Behavior of INPs@BL@Gel

SGF and SIF were prepared according to previously reported protocols [30]. Equal amounts (1 × 10^8^ CFUs) of BL, INPs@BL, and INPs@BL@Gel were incubated in 1 mL of SGF or SIF, respectively. At predetermined time points, aliquots of bacterial suspensions were collected, serially diluted, and plated on TPY agar plates. After incubation at 37°C for 48 h under anaerobic conditions, colony‐forming units were counted to assess bacterial viability.

To evaluate enzyme‐responsive degradation and bacterial release, INPs@BL@Gel was incubated in 2 mL of SIF in the presence or absence of matrix metalloproteinases (MMPs). At designated time points, changes in hydrogel morphology were visually recorded, and the remaining hydrogel mass was measured. Concurrently, released bacterial suspensions were collected, serially diluted, and plated onto TPY agar plates. After 48 h of anaerobic incubation, bacterial colonies were enumerated.

### 4.8 Mucosal Adhesion Capacity of INPs@BL@Gel

INPs were fluorescently labeled, and equal amounts of fluorescently labeled INPs, INPs@BL, and INPs@BL@Gel were co‐incubated with freshly isolated mouse colon tissue at 37°C for 1 h. After incubation, tissues were washed twice with PBS to remove non‐adherent materials and imaged by fluorescence microscopy.

In vivo mucosal adhesion and intestinal retention were further evaluated using a small‐animal fluorescence imaging system (AMI HTX, Spectral Instruments Imaging, USA). DSS‐induced colitis mice (female C57BL/6, 6–8 weeks old, 20–25 g) were randomly assigned to three groups (n = 3) and orally administered equal volumes of INPs, INPs@BL, or INPs@BL@Gel. Fluorescence signals were recorded at predetermined time points in live animals and in excised colon tissues.

All animal experiments were conducted using C57BL/6 mice purchased from the Experimental Animal Center of Xi'an Jiaotong University School of Medicine and were approved by the Ethics Committee of Xi'an Jiaotong University (Approval No. 201929). All procedures were performed in accordance with *the Guide for the Care and Use of Laboratory Animals* and relevant regulations and guidelines. To minimize animal pain and distress, all potentially painful or stressful procedures were conducted under appropriate anesthesia. Throughout the study, trained personnel provided daily care, including food supply and maintenance of appropriate environmental temperature conditions.

ROS‐scavenging ability of INPs@BL@Gel: (1) ABTS radical scavenging assay: The ABTS radical cation (ABTS•^+^) was generated by mixing ABTS solution (7.4 mM) with potassium persulfate (K_2_S_2_O_8_, 2.6 mM) and incubating the mixture in the dark at room temperature for 12–16 h. The resulting ABTS•^+^ solution was then diluted and mixed with INPs@BL@Gel at different concentrations. After incubation, absorbance at 734 nm was measured using a UV–vis spectrophotometer, with anhydrous ethanol serving as the blank control [48]. The ABTS scavenging activity was calculated as: ABTS scavenging capacity (%) = (A_0_ – A)/A_0_ × 100%, where A_0_ is the absorbance of the control, and A is the absorbance of the sample. (2) DPPH radical scavenging assay: A DPPH solution (0.05 mg mL^−1^) prepared in anhydrous ethanol was mixed with INPs@BL@Gel at varying concentrations. After incubation, absorbance at 519 nm was recorded, with anhydrous ethanol as the control [48]. The DPPH scavenging capacity was calculated as: DPPH scavenging capacity (%) = (A_0_ – A)/A_0_ × 100%. (3) Hydroxyl radical (•OH) scavenging assay: The hydroxyl radical scavenging ability was evaluated using a Fenton reaction system. Briefly, INPs@BL@Gel at different concentrations were added to a reaction mixture containing FeSO_4_ (6 mM), salicylic acid (6 mM), and H_2_O_2_ (8.8 mM). The mixture was incubated at 37°C for 30 min, after which the absorbance at 510 nm was measured [49]. The •OH scavenging capacity was calculated as: •OH scavenging capacity (%) = (A_1_ – A_2_)/A_0_ × 100%. (4) Superoxide anion (O_2_•^−^) scavenging assay: INPs@BL@Gel solutions (0.8 mL) at different concentrations were mixed with 0.8 mL of 50 mM Tris‐HCl buffer and incubated at 25°C for 20 min. Subsequently, 0.4 mL of xanthine solution (1.5 mM) was added to initiate superoxide generation. The absorbance at 320 nm was recorded spectrophotometrically [49]. O_2_•^−^ scavenging capacity (%) = (A_1_ – A_2_)/A_0_ × 100%. (5) Intracellular ROS‐scavenging capacity: Human normal colonic epithelial cells (NCM460) were seeded into 96‐well plates and cultured for 24 h. Cells in the control group received fresh medium only, whereas oxidative stress groups were treated with either 400 µM H_2_O_2_ or 50 ng mL^−1^ lipopolysaccharide (LPS). INPs, INPs@BL, or INPs@BL@Gel were then added to the respective wells and incubated at 37°C for 24 h. After treatment, the culture medium was removed and replaced with serum‐free medium containing 10 µM 2′,7′‐dichlorofluorescein diacetate (DCFH‐DA). Cells were incubated at 37°C in the dark for 30 min, followed by three washes with serum‐free medium to remove excess probe. Intracellular ROS levels were subsequently evaluated by laser confocal microscopy and quantified by flow cytometry.

### 4.9 Establishment and Treatment of a DSS‐Induced Colitis‐Associated Mental Disorder Model

Female C57BL/6 mice (6‐7 weeks old, 20–25 g) were acclimated for 7 days prior to experimentation. Colitis‐associated depression was induced by administering drinking water containing 3% (w/v) DSS for 7 consecutive days. Healthy control mice received regular drinking water.

After DSS induction, mice were randomly assigned to four treatment groups (n = 5 per group) and orally administered PBS, INPs, INPs@BL, or INPs@BL@Gel for 5 consecutive days. Following treatment, mice were euthanized, and fecal samples and tissues were collected for subsequent analyses.

### 4.10 Evaluation of BBB Integrity and Permeability TEM

Mice were anesthetized with 10% sodium pentobarbital and transcardially perfused with sterile PBS to remove circulating blood, followed by perfusion with a fixative solution containing 0.25% glutaraldehyde and 4% paraformaldehyde for 5–10 min. Brain tissues from the prefrontal cortex were harvested and further fixed in 2.5% glutaraldehyde and 2% paraformaldehyde at 4°C for 2 h.

Tissues were rinsed with phosphate‐buffered saline, post‐fixed in 1% osmium tetroxide, dehydrated through a graded ethanol series, embedded, sectioned into ultrathin slices, and stained with uranyl acetate and lead citrate. BBB ultrastructure, including endothelial cells, basement membranes, and astrocytic end‐feet, was examined using TEM.

### 4.11 EB Extravasation Assay

BBB permeability was assessed using the EB dye method. Briefly, mice received a tail‐vein injection of 80 µL of 2% (w/v) EB solution. After 1 h, mice were perfused transcardially with PBS to remove intravascular dye. Brain tissues were harvested, photographed, weighed, and homogenized in DMSO. Samples were incubated at 60°C for 24 h to extract EB, followed by centrifugation. The absorbance of the supernatant was measured at 620 nm to quantify EB extravasation.

### 4.12 Behavioral Assessments

All behavioral tests were performed during the dark phase under low‐light conditions (<20 lx). Mouse movements were tracked and analyzed using ANY‐maze 7.3 software. To minimize stress‐induced variability, mice were gently handled daily for three days prior to behavioral testing.

### 4.13 OFT

The OFT was conducted to assess locomotor activity and exploratory behavior [50]. Mice were individually placed in a 50 × 50 × 40 cm open‐field arena and allowed to explore freely for 10 min. Behavioral parameters, including total distance traveled, movement trajectories, and time spent in the central zone, were recorded using infrared night‐vision cameras and analyzed with ANY‐maze 7.3 software. The apparatus was cleaned with 75% ethanol between trials.

### 4.14 EPM

The EPM test was used to evaluate anxiety‐like behavior [50]. The apparatus consisted of two open arms and two closed arms elevated 60 cm above the floor. Mice were placed in the central zone and allowed to explore freely for 10 min. Time spent in open arms, number of open‐arm entries, and locomotor activity were recorded and analyzed using ANY‐maze 7.3. The maze was cleaned with 75% ethanol after each trial.

### 4.15 SPT

The SPT was used to assess anhedonia‐like behavior [50]. Mice were housed individually and provided with two identical bottles containing either 1% sucrose solution or plain drinking water. The positions of the bottles were alternated every 12 h to avoid location bias. After a 24 h food and water deprivation period, sucrose and water intake were measured at 3 and 6 h time points. Sucrose preference was calculated as the ratio of sucrose intake to total fluid intake.

### 4.16 TST

The TST is widely used to evaluate depressive‐like behaviors, including behavioral despair and anhedonia, in experimental animals [50]. Briefly, a 60 cm‐high stand was placed in a quiet testing environment. The distal end of the mouse tail (approximately 1 cm from the tip) was securely attached with adhesive tape, and the opposite end of the tape was fixed to the stand, suspending the mouse in an inverted position with its head maintained at a safe distance above the floor. Each mouse was visually isolated from others using opaque partitions. The total test duration was 6 min, with the first minute defined as an acclimation period during which mice typically exhibit vigorous struggling to adapt to the suspended posture. Immobility time was recorded throughout the remaining 5 min using infrared night‐vision cameras and analyzed with the ANY‐maze 7.3 behavioral analysis system. Upon completion of the test, mice were gently removed from the apparatus, the adhesive tape was detached, and the animals were returned to their home cages.

### 4.17 FST

The FST is a classical behavioral despair paradigm that exploits rodents’ innate aversion to water [50]. When placed in an inescapable aquatic environment, mice initially exhibit active, escape‐oriented behaviors, followed by the gradual emergence of immobility, which is interpreted as a behavioral correlate of despair. For this assay, a 2 L glass beaker was filled with water maintained at approximately 25°C. The water depth was adjusted according to body weight to ensure that the mouse's tail did not touch the bottom of the beaker. Each mouse was gently placed into the water along the wall of the beaker and allowed to swim for a total of 8 min. The first 2 min were considered an acclimation period, and behavioral parameters, including swimming time, immobility time, and climbing time, were recorded during the remaining 6 min. After testing, mice were promptly removed from the water, dried with warm air, and returned to their home cages.

### 4.18 Metagenomic Sequencing and Analysis

Stool samples were submitted to Suzhou Shanjun Biomedical Technology Co., Ltd. (Suzhou, China) for metagenomic analysis. DNA extraction was performed using the MagBeads Fast DNA Kit (MP Biomedicals, Solon, USA), followed by library construction using the KAPA HyperPlus Kit and shotgun sequencing on the MGISEQ‐2000 platform. Raw sequencing reads were quality‐filtered using fastp (v0.23.0), and host‐derived reads were removed by alignment against the GRCm39 reference genome using Bowtie2 (v2.3.5.1). Taxonomic profiling was performed using MetaPhlAn4, while functional pathway annotation was conducted using HUMAnN3 based on KEGG, MetaCyc, and gene family models (GMMs). Alpha diversity indices (Shannon and Simpson) and beta diversity metrics (Bray‐Curtis distance with principal coordinates analysis) were analyzed using the Kruskal‐Wallis test and PERMANOVA, respectively. Differential taxa and metabolic pathways were identified using LEfSe analysis with an LDA score threshold > 2.0. Statistical analyses were performed in R software (v4.3.1) using Wilcoxon rank‐sum and Fisher's exact tests, with false discovery rate (FDR)‐adjusted p values < 0.05 considered statistically significant.

### 4.19 Statistical Analysis

Unless otherwise specified, data are presented as Mean ± SD from three independent experiments. Statistical differences between two groups were analyzed using Student's t‐test, while comparisons among multiple groups were performed using one‐way analysis of variance (ANOVA) followed by Tukey's post hoc test where appropriate. A *p*‐value < 0.05 was considered statistically significant.

## Author Contributions

**Chune Li**: methodology. **Yujie Zhang**: methodology, investigation. **Shunlian Li**: methodology, formal analysis. **Shuo Zhang**: conceptualization, methodology, writing – original draft. **Yiyang Wang**: data curation, validation. **Shaobo Ma**: formal analysis, investigation. **Yudan Zhang**: investigation, data curation. **Songyan Jin**: data curation. **Qiao Li**: investigation, validation. **Xueyong Xie**: validation. **Jiansheng He**: investigation, methodology. **Xiancang Ma**: funding acquisition, supervision, and conceptualization. **Feng Zhu**: supervision, funding acquisition. **Junze Deng**: methodology. **Daocheng Wu**: conceptualization, supervision, writing – review, and editing. **Xueqin Song**: formal analysis. **Qingyan Ma**: validation, formal analysis. **Hang Zhang**: investigation.

## Conflicts of Interest

The authors declare no conflicts of interest.

## Supporting information




**Supporting File**: advs76242‐sup‐0001‐SuppMat.docx.

## Data Availability

The data that support the findings of this study are available from the corresponding author upon reasonable request.
