# Supplementary material for: Multi‐Component Functionalized Bifidobacterium Longum Hydrogel for Multi‐Target Integrated Therapy of Colitis‐Associated Anxiety and Depression
Source: Adv Sci (Weinh). 2026 Jun 22:e76242. Online ahead of print. doi: 10.1002/advs.76242 (PMC13336999; doi:10.1002/advs.76242)
Supplement: Supplementary file 1 — Supporting File: advs76242‐sup‐0001‐SuppMat.docx. [file ADVS-9999-e76242-s001.docx]

**Multi-component functionalized *Bifidobacterium longum* hydrogel for multi-target integrated therapy of colitis-associated anxiety and depression**

*Shuo Zhang^1#^, Yujie Zhang^4#^, Jiansheng He^3#^, Shunlian Li^3^, Qingyan Ma^1^, Qiao Li^3^, Yudan Zhang^2^, Yiyang Wang^2^, Shaobo Ma^1^, Songyan Jin^2^, Chune Li^1^, Xueyong Xie^3^, Hang Zhang^3^, Junze Deng^3^, Xueqin Song^5^, Daocheng Wu^3^*, Xiancang Ma^1,2^*, Feng Zhu^1,2^**

Zhang S^1^, Ma Q^1^, Ma S^1^, Li C^1^, Ma X^1,2^, Zhu F^1,2^

Center for Brain Science & Department of Psychiatry, The First Affiliated Hospital of Xi’an Jiaotong University, Xi’an 710061, China

E-mail: maxiancang@163.com

Zhang Y^2^, Wang Y^2^, Jin S^2^, Ma X^1,2^, Zhu F^1,2^

Center for Translational Medicine & Department of Psychiatry, The First Affiliated Hospital of Xi’an Jiaotong University, Xi’an 710061, China

E-mail: zhufeng1982@xjtu.edu.cn

He J^3^, Li S^3^, Li Q^3^, Xie X^3^, Zhang H^3^, Deng J^3^, Wu D^3^

The Key Laboratory of Biomedical Information Engineering of Ministry of Education, School of Life Science and Technology, Xi’an Jiaotong University, Xi’an, 710049, China

E-mail: wudaocheng@mail.xjtu.edu.cn

Zhang Y^4^

Department of Breast Disease, The Affiliated Cancer Hospital of Zhengzhou University and Henan Cancer Hospital, Zhengzhou, 450008, China

Song X^5^

Biological Psychiatry International Joint Laboratory of Henan, Zhengzhou University, Zhengzhou, 450052, China

^#^ These authors contributed equally to this work

**Experimental Section**

**Preparation of dual-fluorescently labeled INPs:** ICPs were synthesized according to the method described in the main text. After the reaction was complete, the resulting ICPs solution was sonicated for 5 minutes, followed by the addition of 5 μL of Rhodamine B solution (5 mg/mL). Under light-protected conditions, the mixture was magnetically stirred at room temperature for 1 h to allow Rhodamine B to fully label the ICPs surface. Subsequently, the reaction mixture was transferred to a dialysis bag (MWCO = 3.5 kDa) and dialyzed with ddH₂O to remove free Rhodamine B. After dialysis, the liquid in the dialysis bag was freeze-dried to obtain Rhodamine B-labeled ICPs. INPs were synthesized further according to the method described in the main text. The Rhodamine B-labeled ICPs were mixed with FITC-grafted inulin and reacted for 1 h under light-protected conditions. After the reaction, unreacted small molecules and free fluorescent substances were removed by dialysis, followed by freeze-drying to produce dual-fluorescently labeled INPs.

**Contact Time-Dependent Adhesion Test of SG-Gel:** The adhesive properties of SG-Gel were evaluated using a universal testing machine (Sansi Taijie Electrical Equipment Co., Ltd., Zhuhai, China). Fresh porcine colon tissue was rinsed with PBS and cut into pieces of approximately 25 mm × 30 mm. Excess surface liquid was gently removed with filter paper to keep the tissue moist but free of visible droplets. Two pieces of porcine colon tissue were then fixed onto separate glass slides, with the mucosal surfaces facing outward. SG-Gel was uniformly extruded onto the mucosal surface of one tissue piece, with the contact area controlled at 25 mm × 20 mm and the thickness maintained at approximately 2 mm. The other tissue piece was then placed on top of the hydrogel with its mucosal surface facing the SG-Gel, forming a porcine colon tissue/SG-Gel/porcine colon tissue sandwich structure. In the control group, SG-Gel was placed in direct contact with the glass surfaces of two glass slides. Before testing, the load cell was zeroed, and the initial contact position of the sample was recorded. The glass slides were secured with frosted anti-slip tape and mounted onto the grips, ensuring that the adhesive interface remained horizontal and perpendicular to the tensile direction. The adhesion test was performed in a compression–holding–detachment mode. After contact, the samples were held for the preset duration and then vertically detached at a speed of 5 mm/min, while the force–time curves were recorded in real time. Each group was tested at least three times, and a fresh hydrogel contact area was used for each measurement. For the porcine colon tissue group, the tissue surface was re-moistened before each test to minimize the effect of tissue dehydration. The adhesion strength was calculated by dividing the maximum detachment force by the effective contact area.

**Flow cytometric analysis of INP loading on BL:** INPs were synthesized according to the method described in the main text, with 5 μL of the fluorescent dye phycoerythrin (PE, 5 mg/mL) added during the synthesis process for labeling. After the reaction was complete, the resulting solution was transferred to a dialysis bag (MW = 300 kDa) for dialysis to remove free PE. The dialysate was then freeze-dried to obtain PE-INPs. Prior to the experiment, BL was revived and inoculated into TPY liquid medium and cultured to the stationary phase. One milliliter of the BL suspension was taken, centrifuged (4000 rpm, 5 min) to collect the cells, and washed twice with sterile PBS. Subsequently, the bacterial cells were resuspended in 200 μL of PE-INPs solution and gently mixed at room temperature in the dark for 1 h to ensure sufficient contact and binding between PE-INPs and the BL surface, yielding PE-INPs@BL. Concurrently, a control group of BL + free PE was established. Samples were analyzed using a flow cytometer, with fluorescence signals collected via the PE channel. The BL bacterial population was delineated based on the SSC-A scatter plot to exclude debris and obvious aggregates. Subsequently, the PE fluorescence signals of samples in each group were detected. The PE-positive threshold was set based on untreated BL and remained consistent across all samples. At least 10,000 bacterial events were collected for each sample.

**Chromatographic and Mass Spectrometric Conditions:** The mobile phase consisted of 0.01% formic acid in water (phase A) and 1 mM ammonium formate in 95% methanol (phase B). The column temperature was maintained at 45℃, the autosampler temperature at 6℃, and the injection volume was set to 1 μL. Mass spectrometric detection was performed using a 6500 QTRAP+ triple quadrupole mass spectrometer equipped with an electrospray ionization (ESI) source operating in multiple reaction monitoring (MRM) mode. Data acquisition and processing were carried out using Analyst software (v1.7.3, SCIEX) and Biotree Biobud (v2.0.3).

**Targeted Metabolomics Analysis:** Targeted quantitative analyses of short-chain fatty acids (SCFAs) and neurotransmitters were performed by BioQuad Co., Ltd. Fecal samples were transferred into 2 mL EP tubes, vortexed, homogenized using a ball mill for 4 min, and subjected to ultrasonication for 5 min in an ice-water bath, repeated three times. Samples were centrifuged at 5000 rpm and 4℃ to collect the supernatant. Subsequently, 0.1 mL of 50% H_2_SO_4_ and 0.8 mL of extraction solution containing 25 mg/L methyl tert-butyl ether as an internal standard were added. The mixture was vortexed, shaken, sonicated for 10 min (ice-water bath), and centrifuged at 10,000 rpm for 15 min at 4℃. Samples were then stored at -20℃ for 30 min, and the supernatant was transferred to 2 mL glass vials for GC-MS analysis. Serum samples were processed similarly by adding 0.05 mL of 50% H_2_SO_4_ and 0.2 mL of extraction solution containing the internal standard, followed by vortexing, ultrasonication, centrifugation, and cold storage prior to GC-MS analysis. For neurotransmitter quantification, serum and brain tissue samples were extracted using pre-chilled acetonitrile containing 0.1% formic acid. Serum samples (20 μL) were mixed with 80 μL of extraction solvent, vortexed, and sonicated. Brain tissue samples were weighed, combined with copper beads, 80 μL of extraction solvent, and 20 μL of water, followed by repeated grinding and ultrasonication in an ice-water bath. All samples were incubated at -20°C overnight for protein precipitation, centrifuged at 12,000 rpm for 15 min at 4°C, and derivatized with Na_2_CO_3_ and L-BzCl reagent. After the addition of the internal standard (H-std-a-Bz-DF) and centrifugation, supernatants were collected for analysis using an Agilent 1290 Infinity UHPLC system equipped with an ACQUITY UPLC BEH C18 column (1.7 μm, 2.1 mm × 150 mm).

**Supplementary Material 1**

**
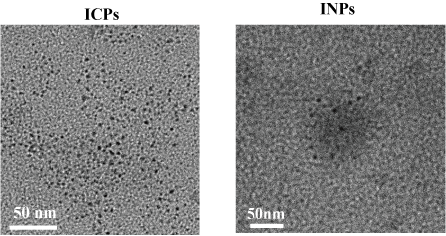
**

**Figure S1.** Representative TEM images of ICPs and INPs. Scale bar: 50 nm.

**
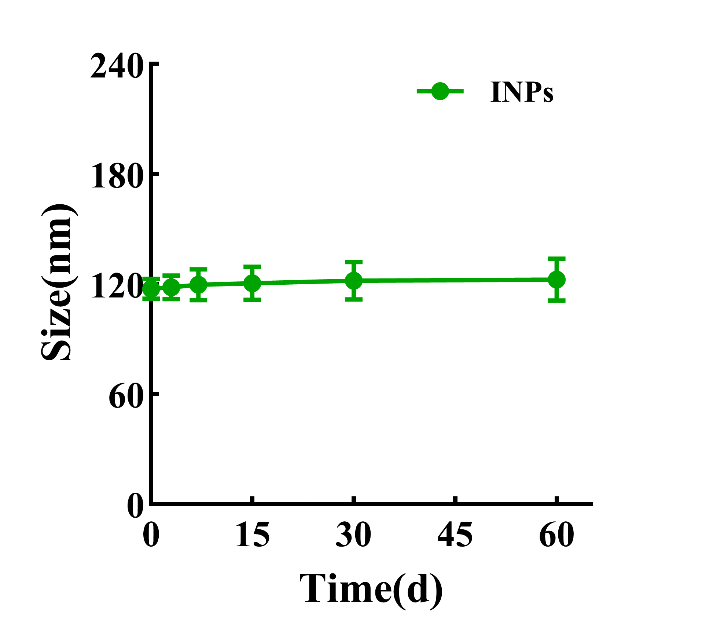
**

**Figure S2.** Particle size changes in INPs over 60 days in a medium containing 10% FBS.


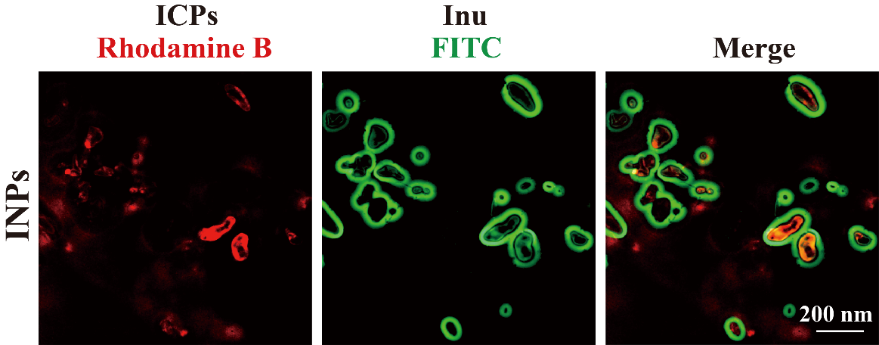


**Figure S3.** Representative CLSM images showing the co-assembly of ICPs and Inu in INPs. ICPs were labeled with Rhodamine B (red), and Inu was labeled with FITC (green). Scale bar: 200 nm.

**
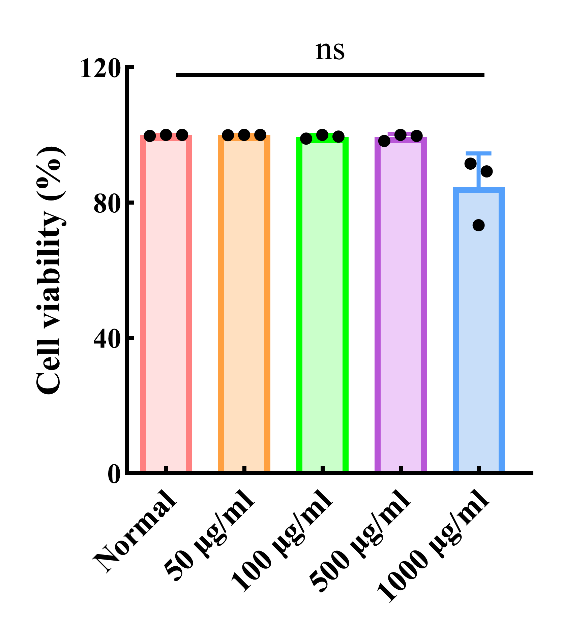
**

**Figure S4.** Cell viability of NCM460 cells after treatment with different concentrations of INPs. n = 3.


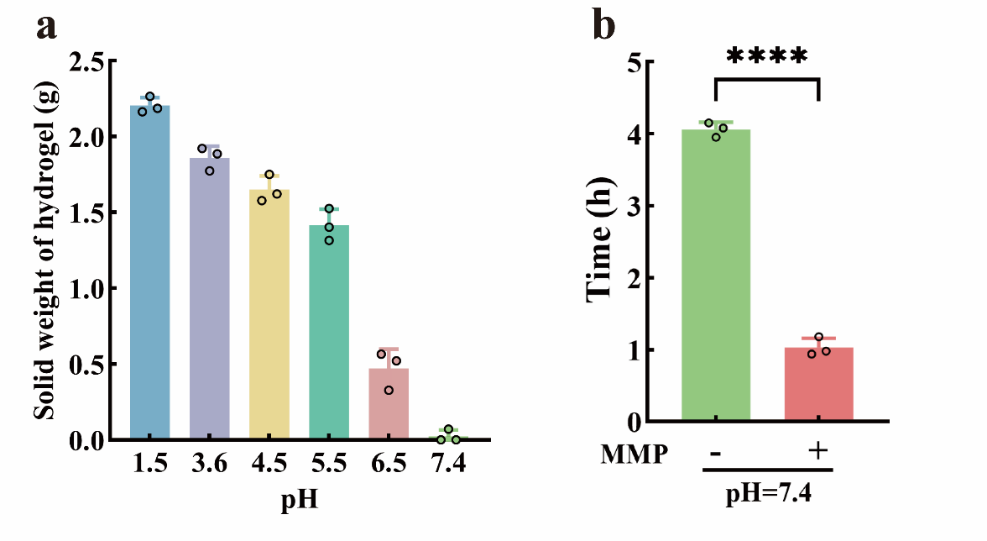


**Figure S5.** The pH- and MMP-Responsive Release from SG-Gel. (a) Change in Residual Solid Weight of SG-Gel After 4 h Under Different pH Conditions. (b) Time required for complete dissolution of SG-Gel at pH = 7.4 in the presence/absence of MMP. Data are expressed as Mean ± SD, with n = 3. Statistical analysis was evaluated with two-tailed Student’s t tests (**p* < 0.05, ***p* < 0.01, ****p* < 0.001, and *****p* < 0.0001).

**
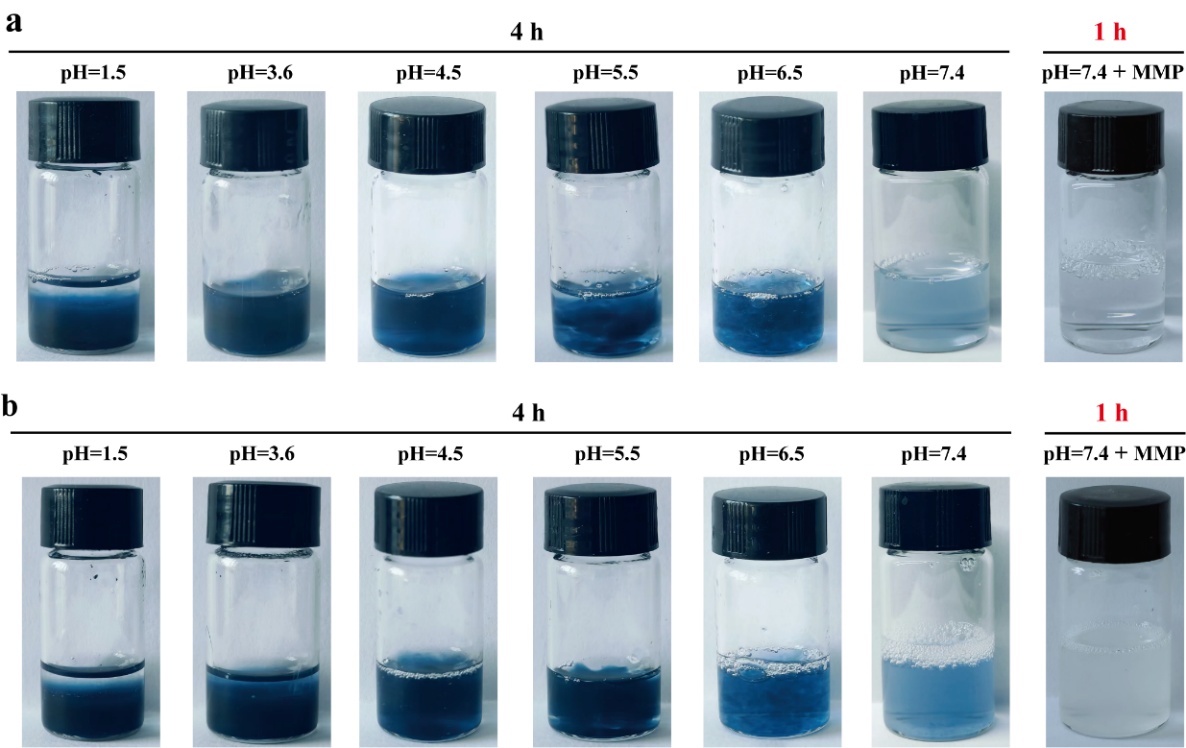
**

**Figure S6.** Duration of pH- and MMP-responsive release from the hydrogel. (a) Representative images of BL@Gel under different conditions, including pH 1.5, 3.6, 4.5, 5.5, 6.5, and 7.4, as well as pH 7.4 with MMP. (b) Representative images of INPs@BL@Gel under different conditions, including pH 1.5, 3.6, 4.5, 5.5, and 6.5, and 7.4, as well as pH 7.4 with MMP.


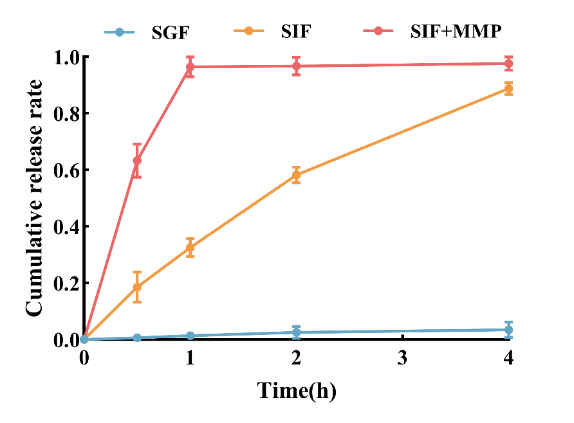


**Figure S7.** Cumulative release profiles of samples over 4 hours in simulated gastric fluid (SGF), simulated intestinal fluid (SIF), and MMP-containing simulated intestinal fluid (SIF + MMP). Data are expressed as Mean ± SD, with n = 3.


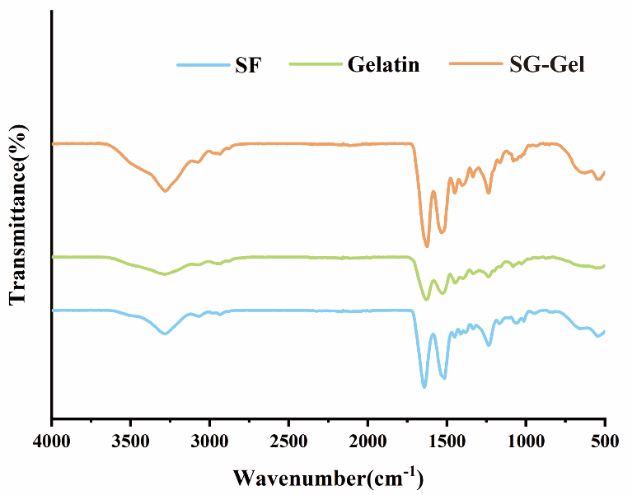


**Figure S8.** FTIR of SF, gelatin and SG-Gel.

**
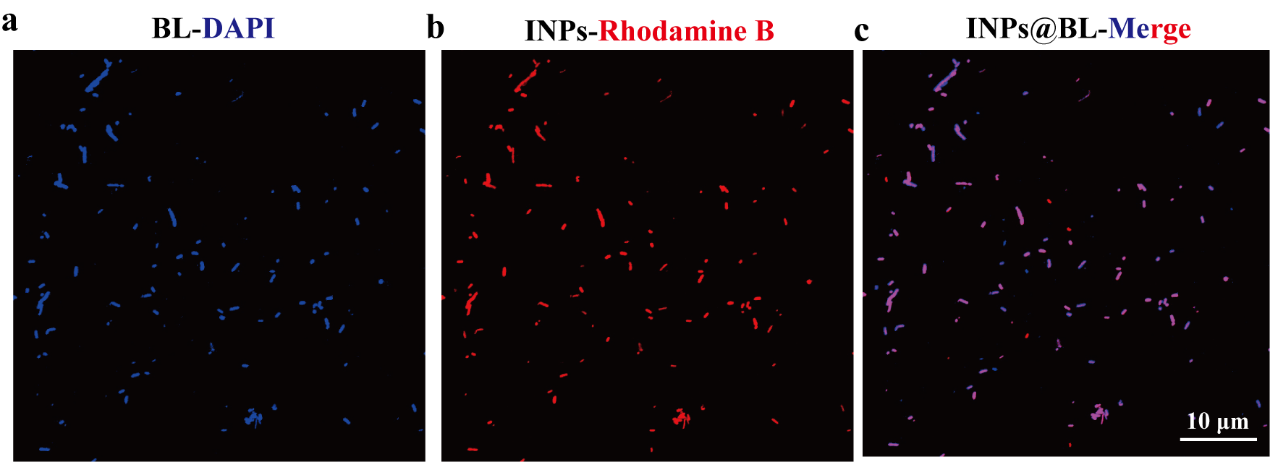
**

**Figure S9.** Representative CLSM images showing the co-assembly of INPs and BL in INPs@BL. (a) DAPI-labeled BL image (blue). (b) Rhodamine B-labeled INPs image (red). (c) Merged image of INPs@BL. Scale bar: 10 μm.

**
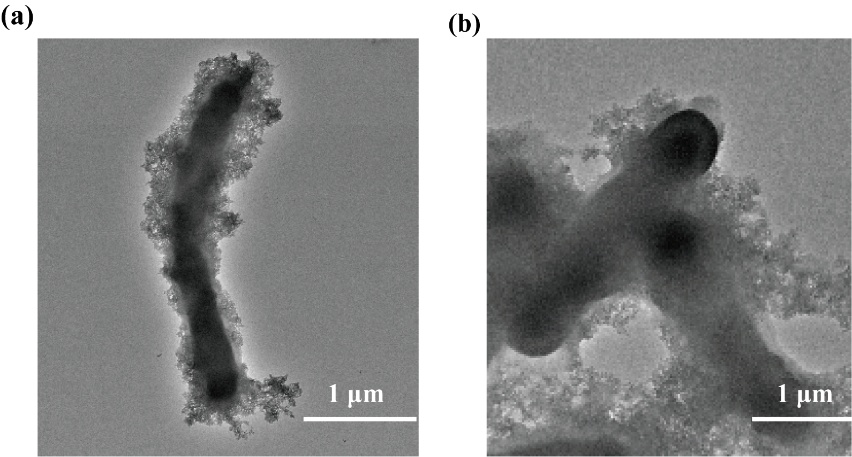
**

**Figure S10.** Representative TEM images of INPs@BL after 4 h of incubation in bile salts (a) and mucin solution (b); scale bar: 1 μm.

**
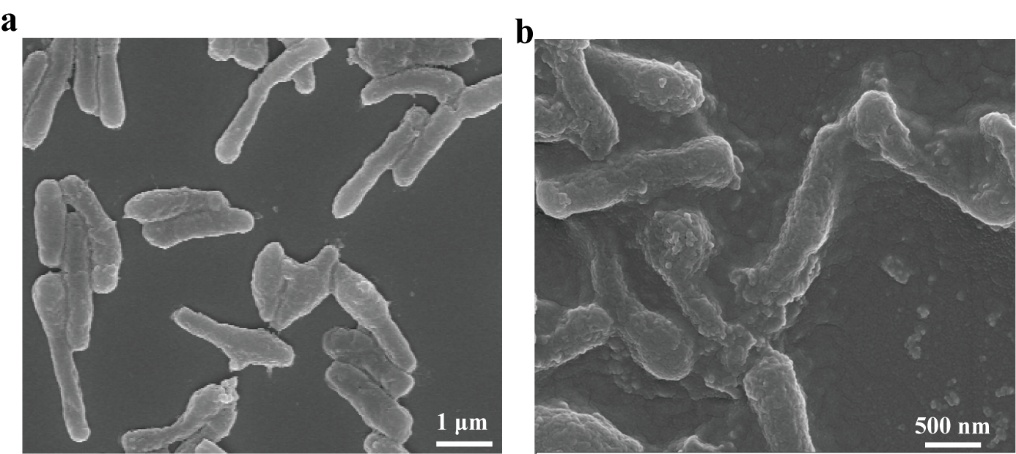
**

**Figure S11.** (a) Representative SEM image of BL, scale bar: 1 μm. (b) Representative SEM image of INPs@BL, scale bar: 500 nm.


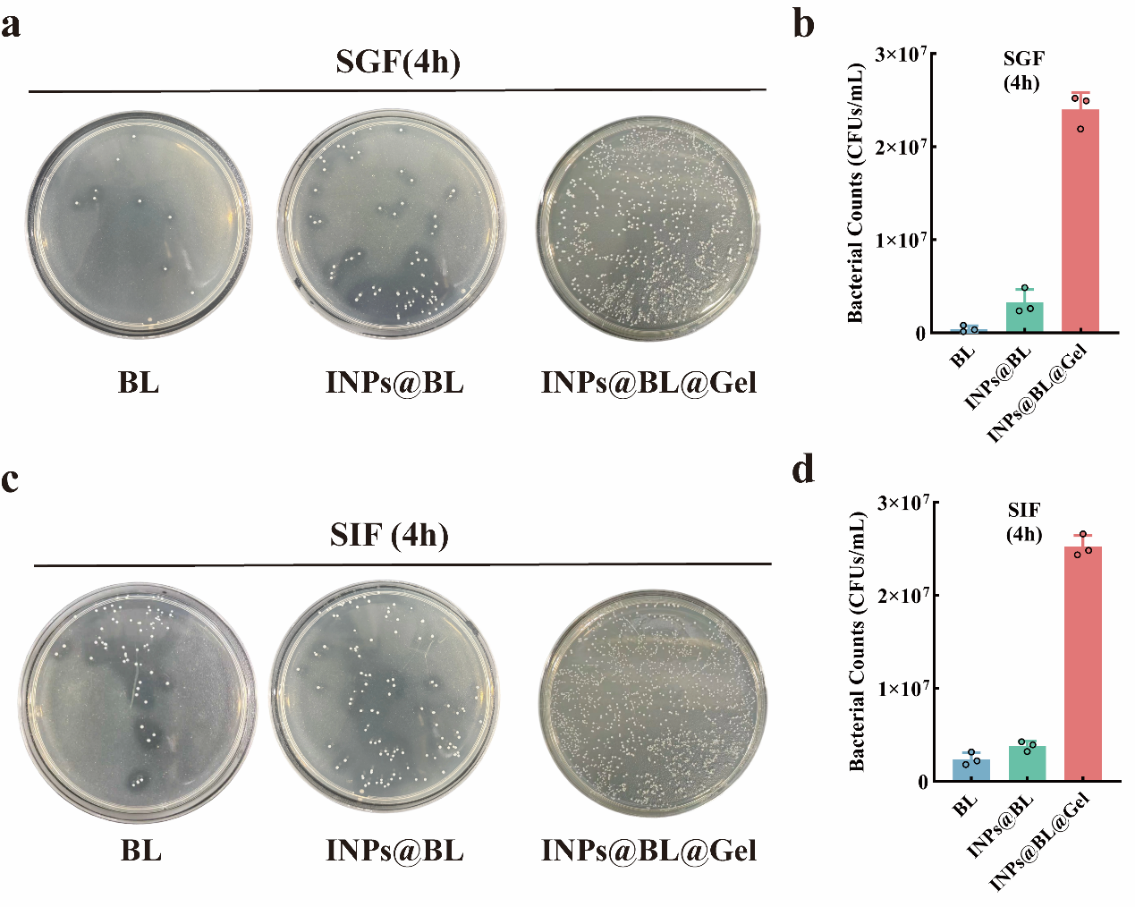


**Figure S12.** Bacterial counts of INPs@BL@Gel after digestion in simulated gastric fluid (SGF) and simulated intestinal fluid (SIF). (a, b) Photographs and corresponding counts of bacterial colonies on TPY agar plates in simulated gastric fluid (SGF); (c, d) Photographs and corresponding counts of bacterial colonies on TPY agar plates in simulated intestinal fluid (SIF). Data are expressed as Mean ± SD, with n = 3.


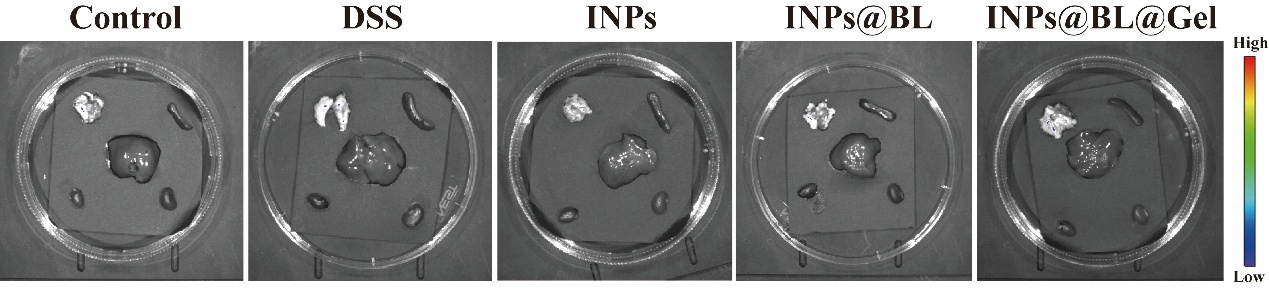


**Figure S13.** Representative fluorescence images of the heart, liver, spleen, lungs, and kidneys in mice from different drug groups labeled with IR780 after oral administration.


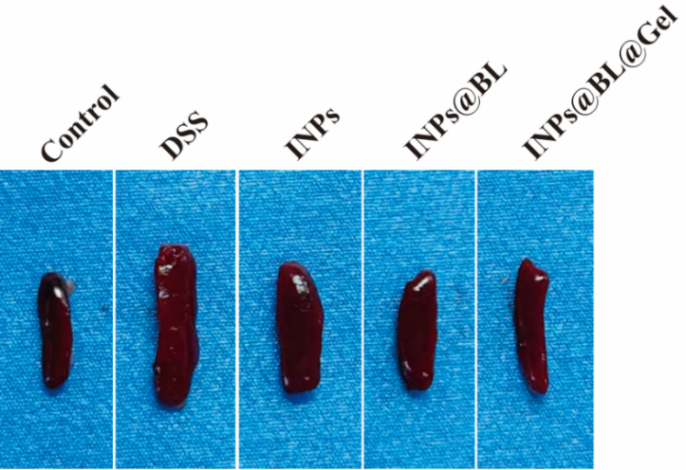


**Figure S14.** Representative images of the spleens of mice in different groups. The INPs@BL@Gel group showed no significant splenomegaly, comparable to the control group.


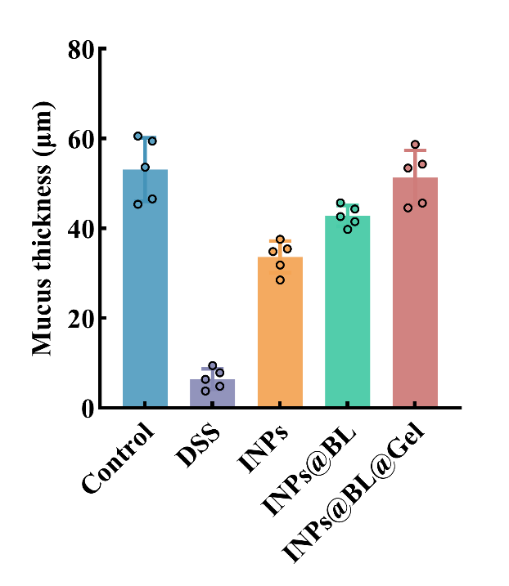


**Figure S15.** Assessment of mucus thickness in the colons of mice in each group by AB-PAS staining. Data are expressed as Mean ± SD, with n = 5.


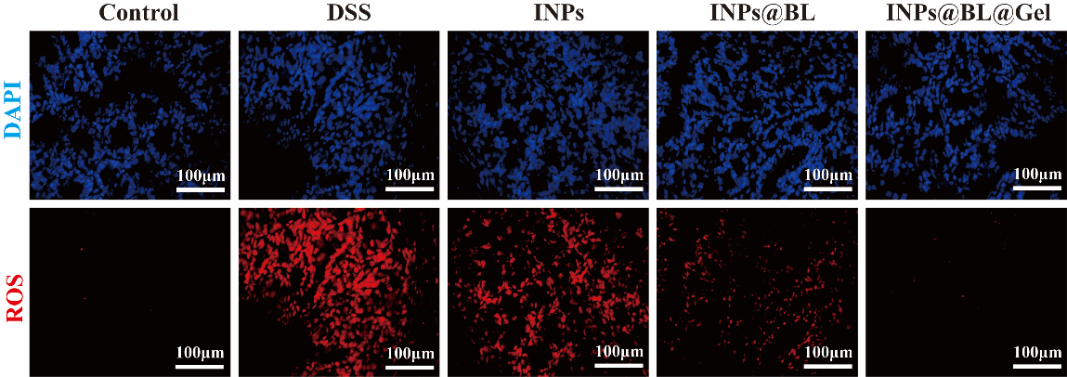


**Figure S16.** Representative ROS immunofluorescence staining images of mouse colons from each group. Cell nuclei were stained with DAPI (blue).


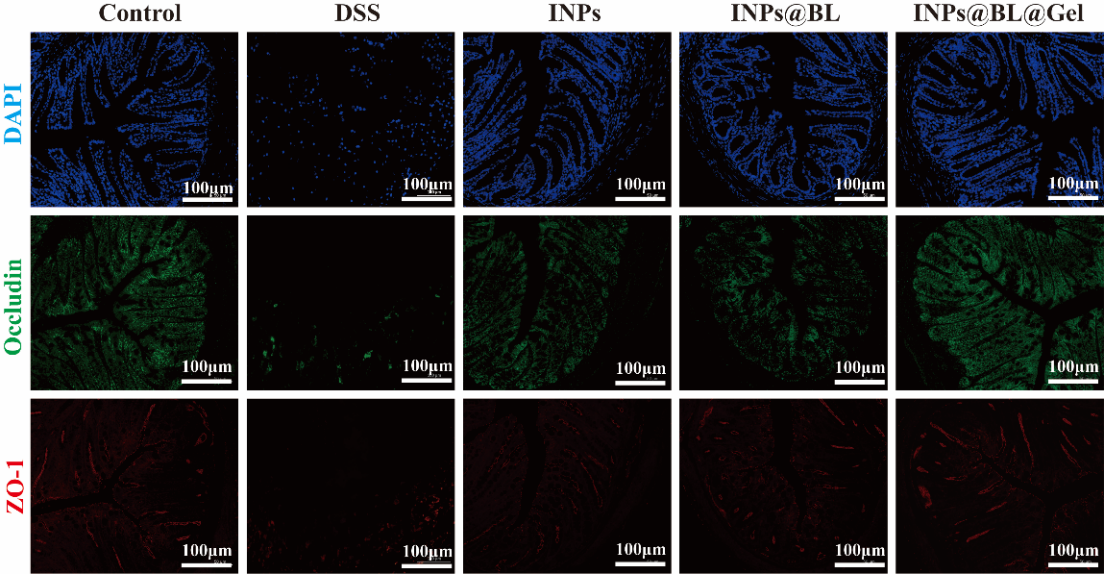


**Figure S17.** Representative immunofluorescence staining images of tight junction proteins ZO-1 (red) and occludin (green) in the colons of each group of mice. Cell nuclei were stained with DAPI (blue).


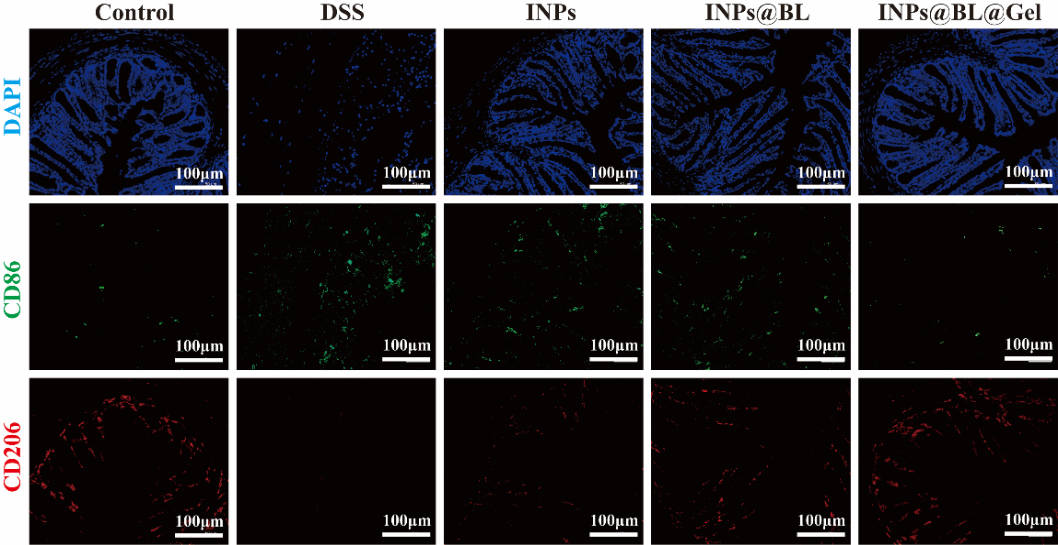


**Figure S18.** Representative immunofluorescence staining images of CD86 (green), a marker for M1 macrophages, and CD206 (red), a marker for M2 macrophages, in the colons of mice from each group. Cell nuclei were stained with DAPI (blue).


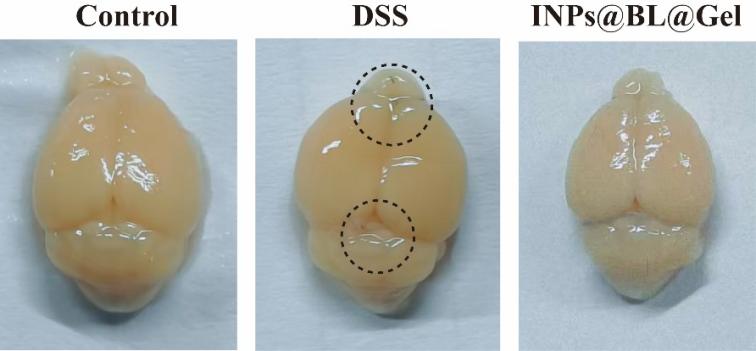


**Figure S19.** Representative images of blood-brain barrier (BBB) permeability in Control, DSS, and INPs@BL@Gel groups assessed via intravenous injection of Evans Blue (EB).


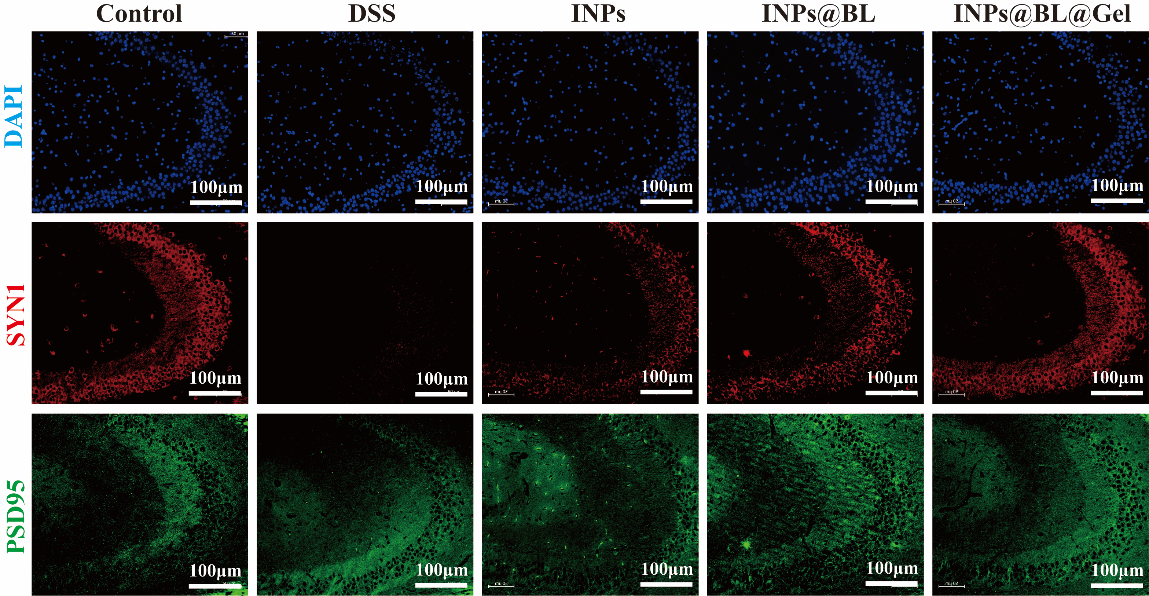


**Figure S20.** Representative immunofluorescence staining images of synapse-associated proteins SYN1 (red) and PSD95 (green) in the hippocampal CA3 region of mice from each group. Cell nuclei were stained with DAPI (blue).


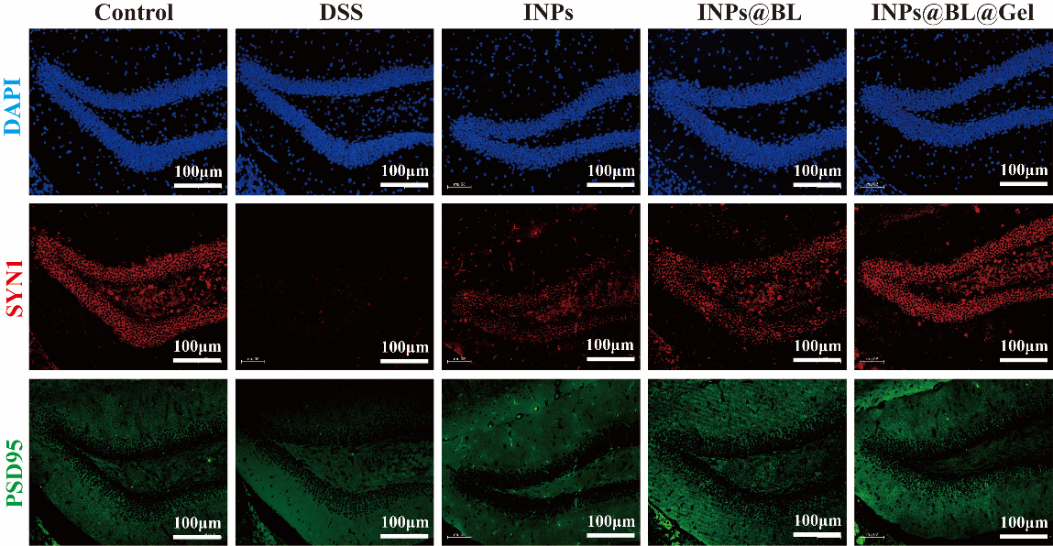


**Figure S21.** Representative immunofluorescence staining images of synapse-associated proteins SYN1 (red) and PSD95 (green) in the hippocampal DG region of mice from each group. Cell nuclei were stained with DAPI (blue).


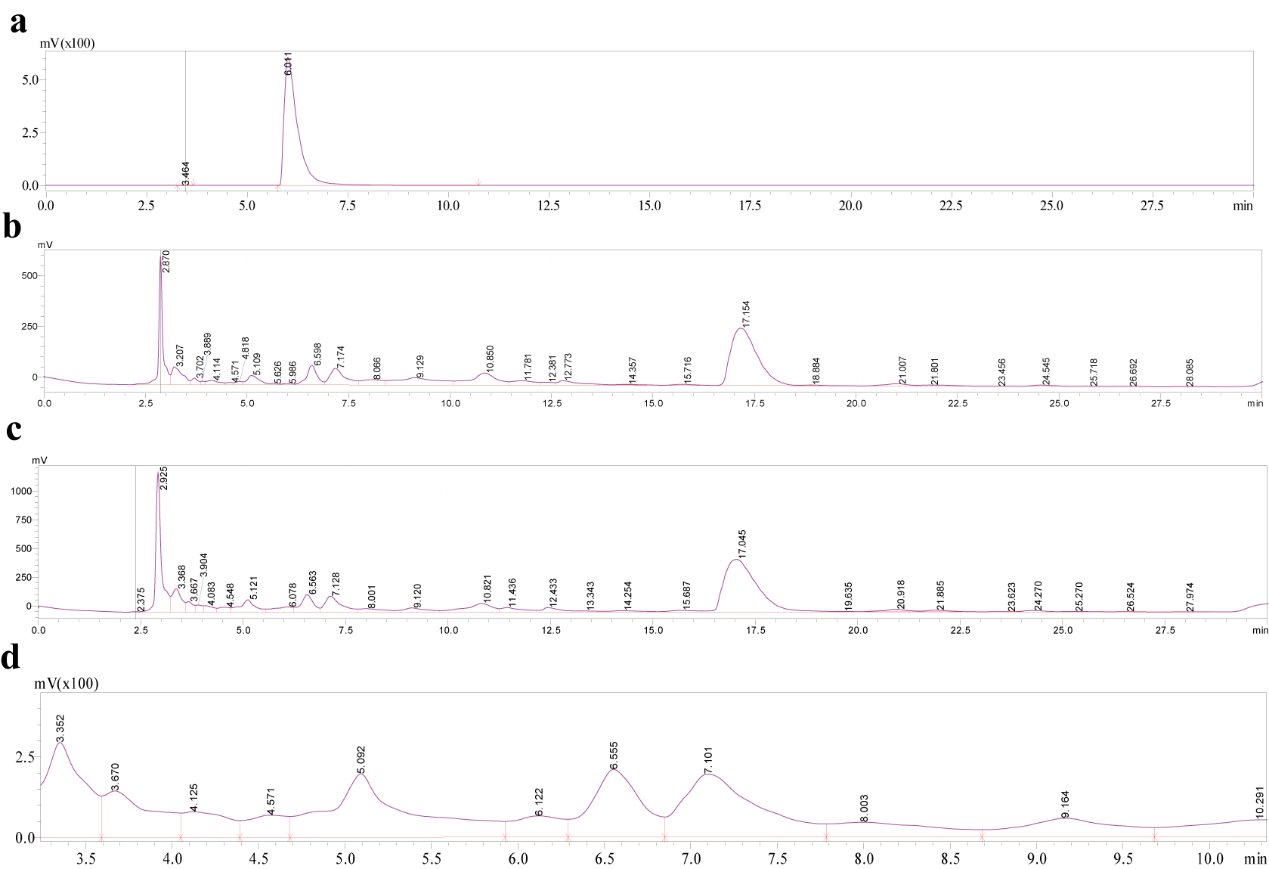


**Figure S22.** HPLC chromatograms of HVA formation under different incubation conditions: (a) HVA standard; (b) Tyr-only incubation group; (c) BL-only incubation group; (d) BL + Tyr co-incubation group.


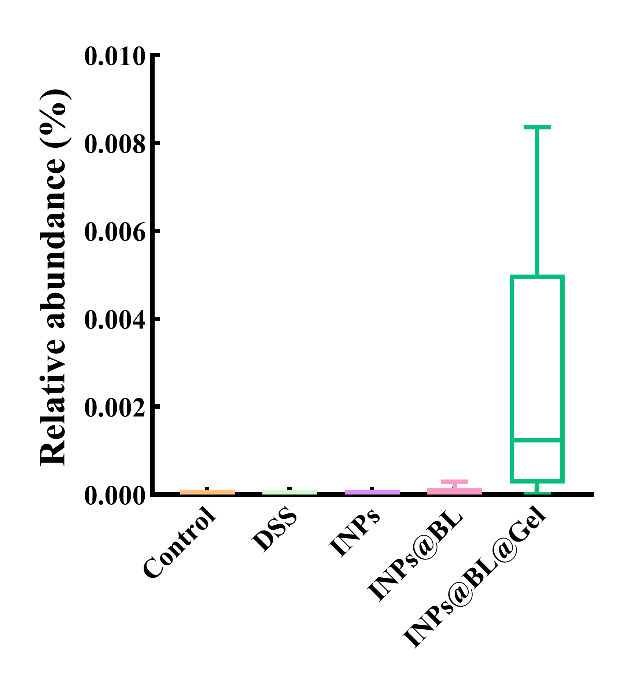


**Figure S23.** Relative abundance of BL in the feces of each group of mice. Data are expressed as Mean ± SD, with n = 5.

**
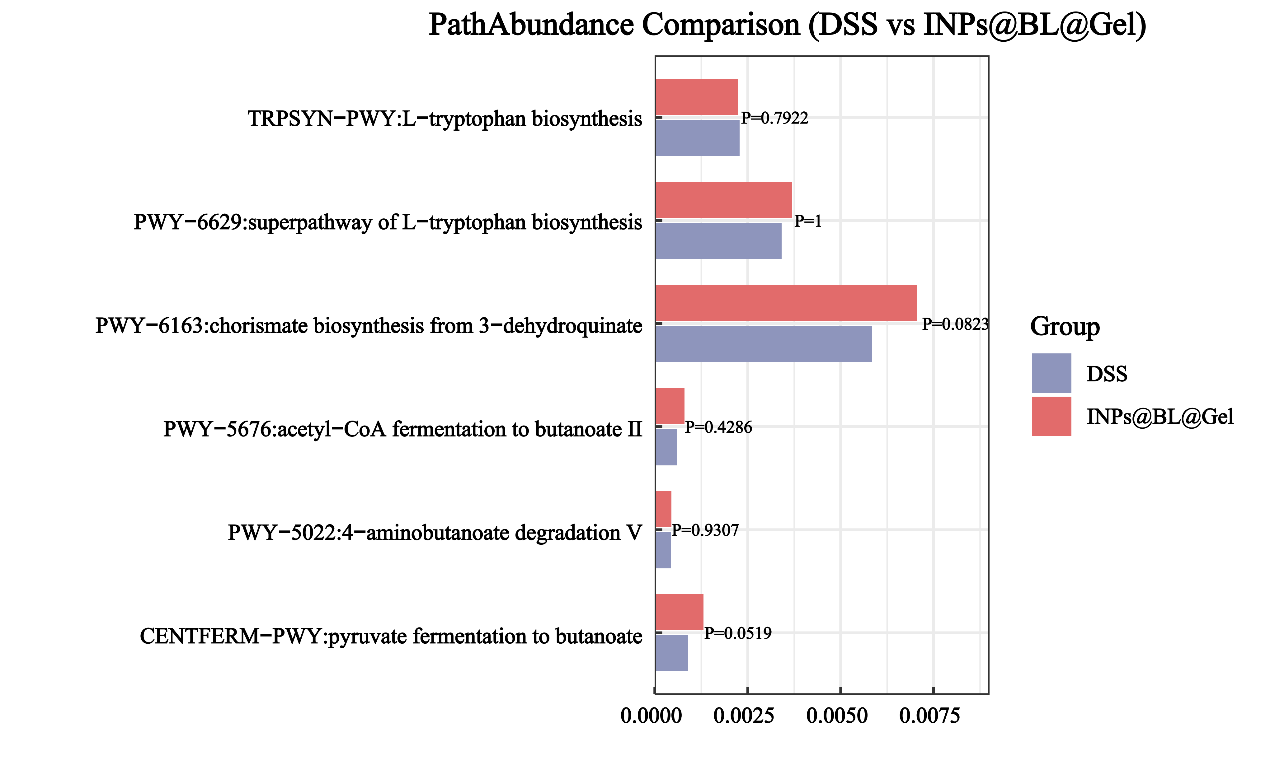
**

**Figure S24.** PathAbundance comparison between the DSS and INPs@BL@Gel groups.

**Supplementary Material 2**

**Table S1.** Optimization of Parameters and Determination of Optimal Conditions for the INPs@BL and SG-Gel Carrier Systems

| Parameter | Tested range | Optimal Value | Description |
| --- | --- | --- | --- |
| Viable bacterial concentration after mixing | 10^7^–10^9^ CFU/200 μL | 10^8^  CFU/200 μL | Concentrations below this value resulted in insufficient therapeutic efficacy, whereas higher concentrations did not further improve efficacy but induced mild inflammation |
| Quality score of SG-Gel | 1%–5% (w/v) | 2.5% (w/v) | A 1% SG-Gel showed insufficient mechanical strength, while a 5% SG-Gel was difficult to inject. The 2.5% SG-Gel provided a balance between protection and operability |
| Volume ratio of INPs@BL to SG-Gel | 1:3-3:1 | 1:1 | At a 1:1 ratio, the bacteria were uniformly distributed, and the release profile exhibited optimal linearity |

**Table S2.** The electron binding energy of coordination atoms in Fe(III)-Bai-ICP NPs and its “preparationraw” materials

| Sample | Binding energy [eV] | | | | | | |
| --- | --- | --- | --- | --- | --- | --- | --- |
|  | O1s | | | Fe 2p 3/2 | | Fe 2p 1/2 | |
| FeCl_3_ | - | | | 711.80 eV | | 725.16 eV | |
| Bai | 529.30 eV | 531.20 eV | - | | - | - | - |
| Fe(III)-Bai ICPs | 531.30 eV | 532.20 eV | 709.10 eV | | 711.58 eV | 721.90 eV | 724.20 eV |

**Table S3.** Scoring system of DAI

| Score | Body weight loss | Stool consistency | Blood |
| --- | --- | --- | --- |
| 0 | ≤1% | Normal | Negative hemoccult |
| 1 | 1-5% | Soft but formed |  |
| 2 | 5-10% | Soft | Positive hemoccult |
| 3 | 10-15% | Very soft |  |
| 4 | >15% | Watery diarrhea | Blood traces in stool visible |

DAI score = (Body weight loss score + Stool consistency score + Blood score) / 3

**Table S4.** Histopathological Assessment of Colitis

| Feature | Score | Description |
| --- | --- | --- |
| Mucosal epithelium | 0 | No mucosa inflammation/prolonged epithelial cells |
|  | 1 | Destruction of barrier/<10% loss of epithelial surface ulcer |
|  | 2 | 10-30% loss of epithelial surface ulcer |
|  | 3 | 30-60% loss of epithelial surface ulcer |
|  | 4 | >60% loss of epithelial surface ulcer |
| Crypt | 0 | No mucosa inflammation/intact crypts |
|  | 1 | Destruction of barrier/<10% loss of crypt sulcer |
|  | 2 | 10-20% loss of crypts ulcer |
|  | 3 | >20% loss of epithelial surface and crypts |
| Cell infiltration and edema | 0 | None |
|  | 1 | Mild Infiltration |
|  | 2 | Moderate Infiltration |
|  | 3 | Severe Infiltration |
| Goblet cells depletion | 0 | Absent |
|  | 1 | Present |

**Table S5.** Nerve injury scores of Brain.

| Degree of injury | Description | Score |
| --- | --- | --- |
| Normal | Cells and extracellular matrix were uninjured | 0 |
| Mildly injured | Slightly swollen cells with decreased protrusions spread in the homogenous extracellular matrix. Pyknotic cells, apoptosis or necrosis was rarely observed | 1 |
| Moderately injured | Oval, lightly stained swollen cells. Increased red neurons, pyknotic cells, apoptosis and necrosis were observed in the granulate matrix adorned with entangled fibers. Neuropils were swollen | 2 |
| Severely injured | Red neurons, pyknotic cells and apoptotic cells were commonly observed. Eosinophilic ghost cells were interspersed in a structureless matrix with disorganized neuropils and tangling fibers | 3 |
| Deadly injured | Deadly injured. Eosinophilic ghost cells and conspicuous coagulative necrosis prevailed. Several pyknotic cells and red neurons interspersed in the cluttered matrix, which was filled with vacuoles left by dead neurons | 4 |
